# An information-theoretic framework for conditional causality analysis of brain networks

**DOI:** 10.1162/netn_a_00386

**Published:** 2024-10-01

**Authors:** Lipeng Ning

**Affiliations:** Brigham and Women’s Hospital, Boston, MA, USA; Harvard Medical School, Boston, MA, USA

**Keywords:** Brain network, Granger causality, State-space representation, Minimum entropy, Spectral factorization

## Abstract

Identifying directed network models for multivariate time series is a ubiquitous problem in data science. Granger causality measure (GCM) and conditional GCM (cGCM) are widely used methods for identifying directed connections between time series. Both GCM and cGCM have frequency-domain formulations to characterize the dependence of time series in the spectral domain. However, the original methods were developed using a heuristic approach without rigorous theoretical explanations. To overcome the limitation, the minimum-entropy (ME) estimation approach was introduced in our previous work (Ning & Rathi, 2018) to generalize GCM and cGCM with more rigorous frequency-domain formulations. In this work, this information-theoretic framework is further generalized with three formulations for conditional causality analysis using techniques in control theory, such as state-space representations and spectral factorizations. The three conditional causal measures are developed based on different ME estimation procedures that are motivated by equivalent formulations of the classical minimum mean squared error estimation method. The relationship between the three formulations of conditional causality measures is analyzed theoretically. Their performance is evaluated using simulations and real neuroimaging data to analyze brain networks. The results show that the proposed methods provide more accurate network structures than the original approach.

## Author Summary

This paper introduces a theoretical framework for causal inference in brain networks using time series measurements based on the principle of minimum-entropy regression. Three types of conditional causality measures are derived based on varying formulations of minimum-entropy regressions. The standard time-domain conditional Granger causality measure is formulated as a special case but with a different expression of the frequency-domain measure. The methods were evaluated using simulations and real resting-state functional MRI data of human brains and compared with standard Granger causality measures and directed transfer functions. Two new formulations of minimum-entropy-based causality measures showed better performance than other methods. The algorithms developed from this work may provide new insights to understand information flow in brain networks.

## INTRODUCTION

Causal inference between multivariate time series is a fundamental problem in data science. It is particularly relevant in neuroimaging research where time series data, such as functional magnetic resonance imaging (fMRI), electroencephalogram (EEG), and magnetoencephalography (MEG), are used to investigate interactions between brain regions that form the so-called brain networks. Brain cognition, function, and memory all involve dynamic and most likely asymmetric interactions between multiple brain regions (Luo et al., 2013; Martínez et al., 2018; Park & Friston, 2013; Sporns, 2013; Tanimizu et al., 2017). Therefore, identification of the directions of information flow in brain networks is critical to understanding the mechanism of brain functions and developing markers for brain diseases (Fitzsimmons, Kubicki, & Shenton, 2013; Fornito, Zalesky, & Breakspear, 2015; Stam, Jones, Nolte, Breakspear, & Scheltens, 2007).

The influence that a brain node exerts over another under a network model is usually referred to as the effective connectivity (EC) (Harrison & Friston, 2003). Several methods have been developed for EC analysis of brain networks, such as dynamic causal modeling (DCM) (Friston, Moran, & Seth, 2013; Stephan et al., 2008), Granger causality measure (GCM) and conditional GCM (cGCM) (Barnett, Barrett, & Seth, 2009; Chen, Bressler, & Ding, 2006; Deshpande & Hu, 2012; Deshpande, LaConte, James, Peltier, & Hu, 2009; Geweke, 1982, 1984; Marinazzo, Liao, Chen, & Stramaglia, 2011; Seth, Barrett, & Barnett, 2015; Solo, 2008; Wang et al., 2020), and directed transfer function (DTF) (Kamiński, Ding, Truccolo, & Bressler, 2001; Kamiński & Blinowska, 1991). DCM uses a deterministic multiple-input and multiple-output system to model the dynamic coupling of the underlying variables. GCM and cGCM characterize the stochastic dependence between time series by quantifying the importance of past values of one variable to the prediction of another. DTF is derived based on a model similar to GCM and cGCM to quantify the dependence of multivariate time series in the spectral domain. More detailed comparisons and explanations of these methods can be found in Friston et al. (2013), Friston et al. (2014), and Kamiński et al. (2001).

The goal of this work is to introduce an information-theoretic framework for causal inference that extends existing GCM and cGCM methods. In this context, several related information-theoretic interpretations or generalizations have already been developed for GCM or cGCM, including transfer entropy (TE) and directed information (DI) (Amblard & Michel, 2011; Barnett et al., 2009; Hlaváčková-Schindler, 2011; Quinn, Coleman, Kiyavash, & Hatsopoulos, 2011; Rissanen & Wax, 1987; Solo, 2008; Vicente, Wibral, Lindner, & Pipa, 2011). Rissanen and Wax (1987) first pointed out the relation between entropy differences and Geweke’s GCM by introducing a causality measure as the difference between the code length for encoding one variable using its own past values and the code length based on the joint past values with other variables. Hlaváčková-Schindler, Paluš, Vejmelka, and Bhattacharya (2007) introduced several methods for causal inference based on mutual information and TE. Hlaváčková-Schindler et al. (2007) and Solo (2008) showed that the GCM between a pair of time series is also a mutual information measure. Barnett et al. (2009) showed the equivalence between GCM and TE for Gaussian processes. The equivalence between GCM and TE has also been extended to generalized Gaussian probability distributions (Hlaváčková-Schindler, 2011), model-free tools for effective connectivity analysis in neuroscience (Vicente et al., 2011), and nonlinear causality detections (Keskin & Aste, 2020). Quinn et al. (2011) applied the DI measure to infer causal relationships in neural spike train recordings based on previous works in Rissanen and Wax (1987). DI is derived from a modified mutual information measure and can be decomposed as the sum of the TE and a term that quantifies the instantaneous coupling (Amblard & Michel, 2011). More detailed overviews about GCM and related information-theoretic formulations can be found in Amblard and Michel (2013) and Seghouane and Amari (2012).

The causality measures introduced in this work are developed based on the minimum-entropy (ME) estimation method proposed in our previous work (Ning & Rathi, 2018). Similar to the classical minimum mean square error (MMSE) approach, the ME estimation method applies a linear dynamic filter to one time series to predict another based on a joint model to minimize the entropy rate of the residual process. However, the ME method uses a different objective function that draws the relationship between the entropy rate, causality measures, and the power spectral density (PSD) functions. Our work (Ning & Rathi, 2018) has shown that the ME method provides new expressions for frequency-domain causality measures that are not provided by TE- or DI-based methods. This work further extends this framework to introduce three types of ME-based causality measures to quantify the conditional causality between multivariate time series and general computation algorithms-based state-space representations. The relationship between the three methods analyzed and their performances are compared using both simulations and in vivo resting-state fMRI (rsfMRI) data of human brains.

## THEORY

### Notations and Background on Granger Causality Measure

Let ***u***_*t*_ = (***x***_*t*_; ***y***_*t*_; ***z***_*t*_) ∈ ℝ^*n*^ denote a zero-mean wide-sense stationary multivariate Gaussian time series, which, for example, represents neural activity measurements in different brain regions measured using neuroimaging techniques. Based on notations as in Solo (2016), without loss of generality, the time series can be jointly modeled by a vector autoregressive (VAR) model

$$G\left(L\right)u_{t}=\epsilon_{t},$$ (1)

which can be decomposed as

$$\left[\begin{array}{ccc} G_{xx}\left(L\right) & G_{xy}\left(L\right) & G_{xz}\left(L\right)\\ G_{yz}\left(L\right) & G_{yy}\left(L\right) & G_{yz}\left(L\right)\\ G_{zx}\left(L\right) & G_{zy}\left(L\right) & G_{zz}\left(L\right) \end{array}\right]\left[\begin{array}{c} x\\ y\\ z \end{array}\right]=\left[\begin{array}{c} \epsilon_{x}\\ \epsilon_{y}\\ \epsilon_{z} \end{array}\right],$$ (2)

where *L* denotes the lag operator such that *L*^*k*^***u***_*t*_ = ***u***_*t*−*k*_ and *G*(*L*) = *I* + *G*_1_*L* + *G*_2_*L*^2^ + … represents the expansion of the VAR fitler. The covariance matrix of the innovation process ***ϵ***_*t*_ is denoted by

$$𝓔\left({\epsilon \epsilon }^{T}\right)=\left[\begin{array}{ccc} \Omega_{xx} & \Omega_{xy} & \Omega_{xz}\\ \Omega_{yz} & \Omega_{yy} & \Omega_{yz}\\ \Omega_{zx} & \Omega_{zy} & \Omega_{zz} \end{array}\right].$$ (3)

In general, the VAR filter *G*(*L*) may have an infinite order represented by a state-space representation model and spectral factorization algorithms (Seth et al., 2015; Solo, 2016). The power spectral density (PSD) function of the time series is equal to

$$S_{u}\left(\theta \right)≔G{\left(e^{i\theta }\right)}^{-1}\Omega G{\left(e^{i\theta }\right)}^{-*},$$ (4)

where (·)^−^* = ((·)^−1^)* for simplicity.

Based on the state-space representation, the subprocess (***x***_*t*_; ***y***_*t*_) can be represented by the following (infinite-order) VAR model using the spectral factorization algorithm (Seth et al., 2015; Solo, 2016)

$$\left[\begin{array}{cc} {\hat{G}}_{xx}\left(L\right) & {\hat{G}}_{xy}\left(L\right)\\ {\hat{G}}_{yx}\left(L\right) & {\hat{G}}_{yy}\left(L\right) \end{array}\right]\left[\begin{array}{c} x\\ y \end{array}\right]=\left[\begin{array}{c} {\hat{\epsilon }}_{x}\\ {\hat{\epsilon }}_{y} \end{array}\right],$$ (5)

where the covariance matrix of ($\hat{\epsilon }$_*x*_; $\hat{\epsilon }$_*z*_)is denoted by

$$𝓔\left(\left[\begin{array}{c} {\hat{\epsilon }}_{x}\\ {\hat{\epsilon }}_{y} \end{array}\right]\left[\begin{array}{cc} {\hat{\epsilon }}_{x}^{T} & {\hat{\epsilon }}_{y}^{T} \end{array}\right]\right)=\left[\begin{array}{cc} {\hat{\Omega }}_{xx} & {\hat{\Omega }}_{xy}\\ {\hat{\Omega }}_{yx} & {\hat{\Omega }}_{yy} \end{array}\right].$$ (6)

Note that the notations in Equations 5 and 6 denoted by $\hat{\cdot }$ are different from those in Equations 2 and 3.

The subprocess ***x***_*t*_ can be further extracted from ***u***_*t*_ or (***x***_*t*_; ***y***_*t*_) and modeled by an infinite-order VAR model using the spectral factorization algorithm, which is represented as

$$A_{xx}\left(L\right)x_{t}=\xi_{x,t},$$ (7)

where ***ξ***_*x*,*t*_ is a white Gaussian process and the one-step-ahead prediction error of ***x***_*t*_ using all its past values with 𝓔(***ξ***_*x*,*t*_$\xi_{x,t}^{T}$) = Σ_*xx*_. The PSD function of ***x*** is equal to

$$S_{x}\left(\theta \right)=A_{xx}^{-1}\left(e^{i\theta }\right)\Sigma_{xx}A_{xx}^{-*}\left(e^{i\theta }\right).$$ (8)

As shown above, spectral factorization is an important technique for modeling multivariate time series, which is also key in Granger causality analysis. For completeness, an overview of the related algorithm and state-space representation are provided in the Supporting Information.

The variable ***ξ***_*x*_ in Equation 7 represents the prediction error of ***x***_*t*_ using all its past values. On the other hand, $\hat{\epsilon }$_*x*_ in Equation 5 or ***ϵ***_*x*_ in Equation 2 represents the prediction error of ***x***_*t*_ using the all the past values of (***x***_*t*_, ***y***_*t*_) or (***x***_*t*_, ***y***_*t*_, ***z***_*t*_), respectively. Since adding more variables reduces the prediction error, the variance of $\hat{\epsilon }$_*x*_ or ***ϵ***_*x*_ is expected to be no larger than that of ***ξ***_*x*_. Based on the difference between the two, the Granger causality measure (GCM) (Geweke, 1982) from ***y*** to ***x*** is defined as

$${𝓕}_{y\to x}=\ln\frac{\det\;\Sigma_{xx}}{\det\;{\hat{\Omega }}_{xx}},$$ (9)

which reflects the extent which the past values of ***y*** improve the prediction of ***x***. The conditional GCM (cGCM) (Geweke, 1984) from the past of ***y*** to ***x*** conditional on all the past value of ***z*** is defined by

$${𝓕}_{z\to x|y}^{\text{Std}}=\ln\frac{\det\;{\hat{\Omega }}_{xx}}{\det\;\Omega_{xx}},$$ (10)

which reflects the additional information in past values of ***z*** that is useful to improve the prediction of ***x***.

### Minimum-Entropy Interpretation of GCM and cGCM

An information-theoretic interpretation of the GCM and cGCM was developed in Ning and Rathi (2018) based on the concept of minimum-entropy (ME) prediction. To introduce this method, consider {***x***_*t*_} as a zero mean multivariate Gaussian time series. The entropy rate per time unit is equal to

$$h\left(\left\{x\right\}\right)=\frac{1}{2}\ln\;\det\;\Sigma_{xx}+\frac{1}{2}n_{x}\left(1+\ln\left(2\pi \right)\right),$$ (11)

$$\;=\frac{1}{4\pi }\int_{-\pi }^{\pi }\ln\;\det\;S_{x}\left(\theta \right)d\theta +\frac{1}{2}n_{x}\left(1+\ln\left(2\pi \right)\right).$$ (12)

For the joint process {***x***; ***y***}, a causal filter *F*(*L*) = $\sum_{k=1}^{\text{inf}}$ *F*_*k*_*L*^*k*^ can be applied to the past values of ***y***_*t*_ to predict ***x***_*t*_ to minimize the entropy rate of the following process

$$x\|y_{t}≔x_{t}-F\left(L\right)y_{t}.$$ (13)

It was shown in Ning and Rathi (2018) that the GCM measure 𝓕_***y***→***x***_ is equal to the entropy difference

$${𝓕}_{y\to x}=2\left(h\left(\left\{x\right\}\right)-h\left(\left\{x\|y\right\}\right)\right).$$ (14)

The conditional causality measure cGCM has a similar formulation as Ning and Rathi (2018):

$${𝓕}_{z\to x|y}^{\text{Std}}=2\left(h\left(\left\{x\|y\right\}\right)-h\left(\left\{x\|yz\right\}\right)\right),$$ (15)

where ***x***‖***yz*** denote the ME prediction error of ***x*** using the past values of ***y*** and ***z***.

There is a closely related information-theoretic interpretation of GCM and cGCM based on transfer entropy (TE) in Barnett et al. (2009), Kaiser and Schreiber (2002), and Schreiber (2000). The major difference between the ME and TE-based interpretations is that the ME approach introduces the residual processes whose PSD functions can be used to derive new frequency-domain GCM (fGCM) and cGCM (fcGCM). Specifically, the ME-based frequency-domain GCM measures fGCM is defined as (Ning & Rathi, 2018)

$${\text{f}}_{y\to x}^{\text{Ent}}\left(\theta \right)=\ln\frac{\det\;S_{x}\left(\theta \right)}{\det\;S_{x\|y}\left(\theta \right)},$$ (16)

whose mean value is equal to 𝓕_***y***→***x***_ based on Equation 12. Moreover, the ME-based fcGCM for ${𝓕}_{z\to x|y}^{\text{Std}}$ is derived in a similar formulation as below:

$${\text{f}}_{z\to x|y}^{\text{Std}-\text{Ent}}\left(\theta \right)=\ln\frac{\det\;S_{x\|y}\left(\theta \right)}{\det\;S_{x\|yz}\left(\theta \right)},$$ (17)

where the mean value over all frequencies is equal to ${𝓕}_{z\to x|y}^{\text{Std}}$.

### Generalized Conditional Causality Measures

**Figure 1.**
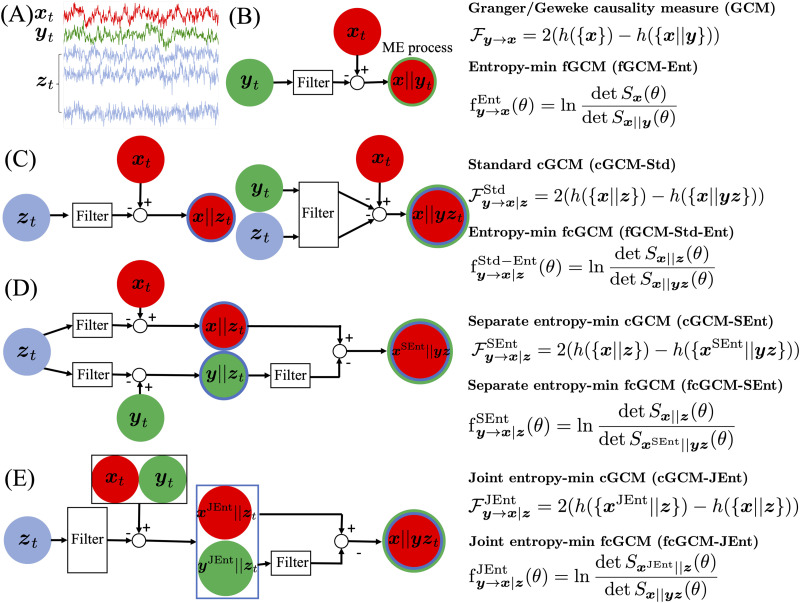
Illustration of the computation algorithms for ME-based causality and conditional causality measures. (A) Illustration of three sets of time series. (B) The method for ME-based GCM from ***y*** to ***x***. (C) The ME-based formulation of the standard cGCM and fcGCM. (D) The algorithm for separate-entropy-minimization-based cGCM and fcGCM. (E) The algorithm for joint-entropy-minimization-based cGCM and fcGCM.

Figures 1B and 1C show the schematic diagrams for the computation procedures for ME-based GCM and cGCM measures. Two filters need to be computed for ${𝓕}_{y\to x|z}^{\text{Std}}$. The first filter illustrated in the left part of 1C removes the past values of ***z*** from ***x***. The second filter in the right part of 1C removes the past values of the joint process (***y***; ***z***) from ***x***. The similarity between ME regression and minimum mean square error (MMSE) estimation motivates different procedures to regress out the dependence of the time series. Consider *x*, *y*, *z* as joint Gaussian random variables to motivate alternative regression procedures. The MMSE estimator for *x* given *y* = *Y*, *z* = *Z* is 𝓔(*x*∣*y* = *Y*, *z* = *Z*), which is also equivalent to

$$𝓔\left(x|y=Y,z=Z\right)=𝓔\left(\left(x|z=Z\right)|\left(y|z=Z\right)=Y\right),$$ (18)

which are equivalent representations for the mean of the marginal distribution of *x* given *y* = *Y*, *z* = *Z*. The above equality indicates that the MMSE estimation conditional on two variables can be equivalent computed in two steps by taking the conditional expectations on the two variables separately. It is interesting to note that the equivalence between the two formulations does not generalize to the case of the ME estimations. Unlike the MMSE estimation problem, the residual process by applying the ME filter to a joint process differs from the result obtained by regressing each subprocess separately. By changing the orders of the ME filtering procedures for joint or sub-processes, we introduce two new formulations for conditional causality measures, as illustrated in Figures 1D and 1E. More details of the computation procedures are explained in the following subsections.

#### The separate entropy minimization approach.

For the method illustrated in Figure 1D, two filters are applied to regress out ***z*** from ***x*** and ***y***, to compute the corresponding ME residual processes, denoted by ***x***‖***z*** and ***y***‖***z***, respectively. Then, the third filter is applied to regress out ***y***‖***z*** from ***x***‖***z*** to obtain the ME residual process, which is defined as

$$x^{\text{SEnt}}\|yz≔\left(x\|z\right)\|\left(y\|z\right).$$ (19)

Then, we introduce the corresponding cGCM, defined as

$${𝓕}_{y\to x|z}^{\text{SEnt}}≔{𝓕}_{y\|z\to x\|z},$$ (20)

$$\;=2\left(h\left(\left\{x\|z\right\}\right)-h\left(\left\{x^{\text{SEnt}}\|yz\right\}\right)\right),$$ (21)

where SEnt represents *separate entropy minimization*. The corresponding frequency-domain fcGCM is given by

$${\text{f}}_{y\to x|z}^{\text{SEnt}}\left(\theta \right)≔{\text{f}}_{y\|z\to x\|z}^{\text{Ent}}\left(\theta \right),$$ (22)

$$\;=\ln\frac{\det\;S_{x\|z}\left(\theta \right)}{\det\;S_{x^{\text{SEnt}}\|yz}\left(\theta \right)}.$$ (23)

To compute ${𝓕}_{y\to x|z}^{\text{SEnt}}$, the first step is to derive the two ME residual processes ***x***‖***z*** and ***y***‖***z***, which can be obtained using the spectral factorization algorithm similar to the computation of 𝓕_***x***‖***y***_. The next step is to model the joint process (***x***‖***z***; ***y***‖***z***), which requires a nontrivial augmented state-space representation. More details about the algorithm and an equivalent simplified formulation are provided in the Supporting Information.

#### The joint entropy minimization approach.

Figure 1E illustrates another formulation of conditional causality measures. Different from Figure 1D, the first step in Figure 1E is to regress out the past values of ***z*** from the joint process (***x***; ***y***) with the ME residual process denoted by (***x***^JEnt^‖***z***; ***y***^JEnt^‖***z***), which is different (***x***‖***z***; ***y***‖***z***), where superscript JEnt represents *joint entropy minimization*. Next, the second ME filter is applied to regress out the past values of ***y***^JEnt^‖***z*** from ***x***^JEnt^‖***z*** to obtain the residual process, which turns out to be equal to ***x***‖***yz***, that is,

$$x\|yz=\left(x^{\text{JEnt}}\|z\right)\|\left(y^{\text{JEnt}}\|z\right).$$ (24)

We define the corresponding cGCM as

$${𝓕}_{y\to x|z}^{\text{JEnt}}≔{𝓕}_{y^{\text{JEnt}}\|z\to x^{\text{JEnt}}\|z},$$ (25)

$$\;=2\left(h\left(\left\{x^{\text{JEnt}}\|z\right\}\right)-h\left(\left\{x\|yz\right\}\right)\right).$$ (26)

The frequency-domain cGCM is defined accordingly as

$${\text{f}}_{y\to x|z}^{\text{JEnt}}\left(\theta \right)≔{\text{f}}_{y^{\text{JEnt}}\|z\to x^{\text{JEnt}}\|z}\left(\theta \right),$$ (27)

$$\;=\ln\frac{\det\;S_{x^{\text{JEnt}}\|z}\left(\theta \right)}{\det\;S_{x\|yz}\left(\theta \right)},$$ (28)

whose mean value is equal to ${𝓕}_{y\to x|z}^{\text{JEnt}}$.

To compute ${𝓕}_{y\to x|z}^{\text{JEnt}}$ and ${\text{f}}_{y\to x|z}^{\text{JEnt}}$(*θ*), the model for (***x***^JEnt^‖***z***; ***y***^JEnt^‖***z***) and ***x***^JEnt^‖***yz*** can be derived analytically using the joint state-space representation. Therefore, computation complexity is much lower than those required by the standard cGCM and the separate entropy minimization method since no spectral factorization algorithms are needed. More details about the analytical expressions for ${𝓕}_{y\to x|z}^{\text{JEnt}}$ and ${\text{f}}_{y\to x|z}^{\text{JEnt}}$(*θ*) are provided in the Supporting Information.

### On the Relationship Between the Three cGCM Measures

As explained in Equation 18, the three ME-based cGCM measures are motivated by equivalent formulations for MMSE estimation methods. However, the three cGCM measures have different values. A theoretical relationship between the three measures is provided in the following proposition.

**Proposition 1.** *For a joint zero-mean wide-sense stationary Gaussian process* (***x***; ***y***; ***z***)*, the* ${𝓕}_{y\to x|z}^{\text{Std}}$, ${𝓕}_{y\to x|z}^{\text{SEnt}}$, ${𝓕}_{y\to x|z}^{\text{JEnt}}$ *measures defined in* *Equations 15*, *21**, and* *26**, respectively, satisfy that*

$${𝓕}_{y\to x|z}^{\text{JEnt}}\geq {𝓕}_{y\to x|z}^{\text{Std}}\geq {𝓕}_{y\to x|z}^{\text{SEnt}}\geq 0.$$ (29)

The inequality between ${𝓕}_{y\to x|z}^{\text{JEnt}}$ and ${𝓕}_{y\to x|z}^{\text{Std}}$ reflects the difference between ***x***^JEnt^‖***z*** and ***x***‖***z***. The entropy of ***x***^JEnt^‖***z*** is higher than that of ***x***‖***z***, which has the minimum entropy among all processes of the form ***x***_*t*_ − *F*(*L*)***z***_*t*_. The joint ME method may be better for preserving the dependence between ***x*** and ***y*** to avoid over-regression of ***z***. On the other hand, the inequality between 𝓕^Std^ and 𝓕^SEnt^ reflects the entropy difference between ***x***^SEnt^‖***yz*** and ***x***‖***yz***. In the first step for computing ***x***^SEnt^‖***yz***, the two filters may over regress ***z*** in the computation of ***x***‖***z*** and ***y***‖***z***, leading to a higher entropy than ***x***‖***yz***. Therefore, compared with the standard method ${𝓕}_{y\to x|z}^{\text{Std}}$, ${𝓕}_{y\to x|z}^{\text{JEnt}}$ involves more conservative repression of ***z*** while ${𝓕}_{y\to x|z}^{\text{SEnt}}$ involves more aggressive regression of ***z***. Again, it is noted that all three methods are motivated based on equivalent MMSE formulations. Thus, none of the three methods should be considered to be incorrect solutions based on the proposed formulations. The following proposition shows that three methods are equivalent for detecting meaningful causal dependence based on the joint model in Equation 2.

**Proposition 2.** *Consider the VAR-type model for joint process* (***x***; ***y***; ***z***) *in* *Equation 2**; then* ${𝓕}_{y\to x|z}^{\text{JEnt}}$ = ${𝓕}_{y\to x|z}^{\text{Std}}$ = ${𝓕}_{y\to x|z}^{\text{SEnt}}$ = 0 *if and only if G_xy_*(*L*) = 0.

The proofs for both propositions are provided in the Supporting Information. In practice, the joint model 2 is usually estimated based on a finite segment of noisy measurements. Therefore, it is unlikely that an entry of the model is strictly zeros. In this case, the sensitivity of the three cGCM measures to modeling error or measurement noise is more important than their relative values to correctly detect causal relationships between time series and reject false positive results. To this end, we will compare the performance of the three cGCM measures using simulations and real data in the next section.

## METHODS

### Simulations

**Figure 2.**
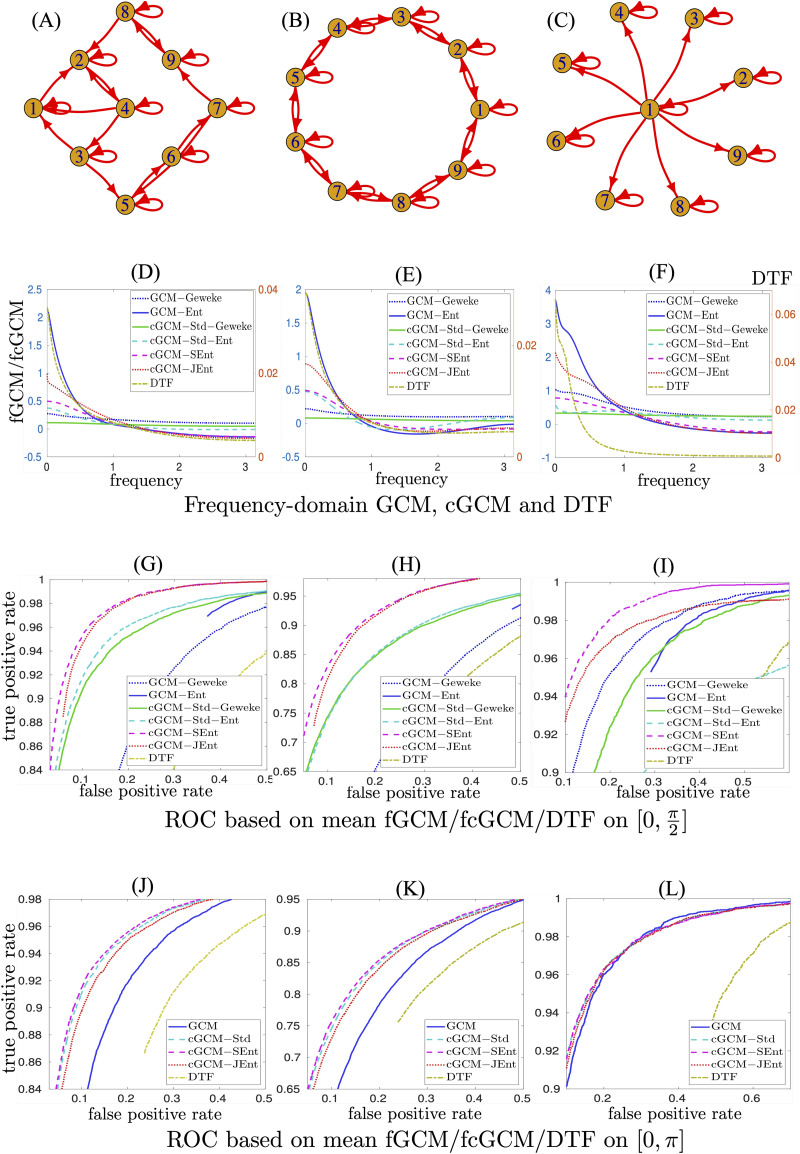
Illustration of simulation results corresponding to VAR-type models. The first row demonstrates the structure of three VAR-type models used in the simulations. The second row shows the sample mean of frequency-domain GCM, cGCM, and DTF functions for all nonzero connections in the first row. The third row shows the ROC curves based on mean fGCM and fcGCM values in the frequency interval [0, $\frac{\pi }{2}$]. The last row illustrates the ROC curve based on time-domain GCM and cGCM measures, which are the mean values in [0, *π*].

The sensitivity and specificity of the three cGCM and GCM measures were compared using simulation data based on three different network structures. The simulated time series were generated based on three VAR models of the following form:

$$u_{t+1}={Au}_{t}+\epsilon_{t},$$ (30)

where the covariance of the process ***ϵ*** had a compound symmetry structure given by

$$𝓔\left(\epsilon_{i,t}\epsilon_{j,t}\right)=\left\{\begin{array}{ll} 1 & \text{if}\;i\neq j,\\ 2 & \text{if}\;i=j, \end{array}\right.$$ (31)

to represent correlated innovation processes. Three sets of system matrices *A* were simulated according to the graphs illustrated in Figures 2A, B, and C, respectively, where the entry *A*_*ij*_ = *a* > 0 if there is a directed connection from node *j* to node *i*, including the case when *i* = *j*. The graph in Figure 2A has the same topology as an example used in the MVGC MATLAB toolbox (Barnett & Seth, 2014). Figure 2B has a circular structure. Figure 2C illustrates a star-shaped graph where all nodes on the circular boundary receive input from the center node. For each VAR model, time series data were simulated with *a* being chosen such that the maximum magnitude of the eigenvalues of *A* were equal to 0.85.

In the experiments, 1,000 independent simulations were generated based on the VAR models, with the length of observation being 120 for Figures 2A and 2B and 60 for Figure 2C, which is shorter than typical fMRI measurements in clinical studies. The simulated data were used to estimate the VAR model parameters using least-square fitting methods. The VAR models were further transformed into state-space representations that were applied to compute the GCM and cGCM measures using the algorithms provided in the Supporting Information. For comparison, the directed transfer function (DTF) method (Kamiński & Blinowska, 1991) was also applied to estimate the frequency-dependent measures of directed connections between time series.

Random permutations were implemented following the approach in Barnett and Seth (2014), where the significance of the estimated values was assessed based on the null distribution of the GCM, cGCM, and DTF. To generate the null distribution for the causality measures from the *i*th node to the other nodes, only the time index of the *i*th time series is randomly permutated while keeping the other time series unchanged. A total of 1,000 random permutations were applied for each time series to generate the null distributions for each pair of GCM, cGCM, or DTF measures in both the time domain and the frequency domain. Thus, 36,000 (i.e., 1, 000 × 9 × 8/2) permutations were generated in each trial. A connection was declared if the *p* value of the non-permuted measures in the null distribution was lower than a preselected significance level. A varying threshold for the significance level was applied to classify the causality measures to compute the false-positive rate (FPR) and true-positive rate (TPR). The relationship between FPR and TPR provides the receiver operating characteristic (ROC) curve to examine the trade-off between sensitivity and specificity of the causality measures.

Note that the model coefficient *a* is positive, which makes the VAR filters low-pass in the frequency domain. To examine the impact of high-pass filters, another set of experiments with negative coefficients and the same network structures are presented in the Supporting Information. Moreover, the Supporting Information also includes another simulation example that compares the GCM and DTF measures in a basic network structure with only two nodes.

### In Vivo rsfMRI Analysis

The performance of these ME-based cGCM algorithms and standard methods were compared using in vivo MRI data from 100 unrelated subjects of the Human Connectome Project (Glasser et al., 2013; Smith et al., 2013). Each subject has four rsfMRI scans with 2-mm isotropic voxels, matrix size = 104 × 90, TE = 33.1 ms, and TR = 0.72 s, which have been processed by the minimal processing pipeline (Smith et al., 2013). Moreover, each subject has two diffusion MRI (dMRI) scans that were registered with the *T*_1*w*_ MRI data. The dMRI data have 1.25-mm isotropic voxels with matrix size of 210 × 180 and 111 slices. Moreover, the dMRI data include three nonzero b-values at *b* = 1,000, 2,000, and 3,000 s/mm^2^ with TE = 89 ms and TR = 5.5 s (Sotiropoulos et al., 2013).

The Automated Anatomical Labeling (AAL) atlas (Tzourio-Mazoyer et al., 2002) was applied to separate the brain and cerebellum into 120 regions. Then, the mean rsfMRI time series from each brain region is extracted and further processed to remove the mean signal and normalize the standard deviation. The proposed time- and frequency-domain GCM and cGCM methods were applied to analyze the 120-dimensional rsfMRI data, similar to the frequency-domain brain connectivity analysis used in Cassidy, Rae, and Solo (2015). Both the Akaike information criterion (AIC) and the Bayesian information criterion (BIC) (McQuarrie & Tsai, 1998) have been used for model selection. A first-order VAR-type model was optimal based on both AIC and BIC. The estimated VAR model parameters were applied to estimate the GCM, cGCM, and DTF measures. The cGCM measure between two brain regions was computed conditional on the other 118-dimensional time series from all other regions. In contrast, the GCM measures were estimated based on a linear model of the two-dimensional time series. All the measures were computed based on the same VAR-type model of the 120-dimensional time series to ensure the comparisons were consistent as suggested in Barnett, Barrett, and Seth (2018), Barnett and Seth (2015), and Dhamala, Liang, Bressler, and Ding (2018). The frequency-domain GCM, cGCM, and DTF measures were computed as the average value in the frequency interval between 0.01 and 0.1 Hz. Similar to the time-domain GCM, the time-domain DTF was computed as the average value in the entire frequency domain.

For human brains, the ground true effective connections between brain regions are unknown. But the structural connectivity (SC) via white matter pathways provides the biological substrates for brain effective connectivity (Park & Friston, 2013; Sokolov et al., 2018, 2019). To this end, the multifiber tractography algorithm developed in Malcolm, Shenton, and Rathi (2010) and Reddy and Rathi (2016) was applied to the diffusion MRI data to estimate the whole-brain fiber bundles. Then, the anatomically curated white matter atlas (Zhang et al., 2018) was applied to the tractography results to filter out possibly false connections. Next, the percentage of fiber bundles between each pair of ROIs was computed, which was considered as the weight of the SC.

## RESULTS

### Results on Simulation Experiments

The second row of Figure 2 illustrates the sample mean fGCM, fcGCM (left y-axis), and the DTF function (right y-axis) of all nonzero connections corresponding to the three network structures illustrated in the first row of Figure 2. The ME-based fGCM and fcGCM functions have negative values at the high-frequency range. On the other hand, the original fGCM, fcGCM, and DTF functions have positive values.

The third row of Figure 2 illustrates the ROC curves, that is, TPR versus FPR, when the average values of fGCM and fcGCM functions at [0 $\frac{\pi }{2}$] frequency interval were used to detect connections. The abbreviations cGCM-Std, cGCM-SEnt, and cGCM-JEnt represent the standard cGCM (Equation 15) and separate and joint ME estimation-based cGCMs, respectively. In all three figures, cGCM-SEnt has the highest TPR at a fixed FPR value. The interval [0 $\frac{\pi }{2}$] is chosen heuristically to capture low-frequency information since the underlying VAR filters are low-pass. A more accurate selection of the frequency range is potentially useful to further improve the performance.

The last row of Figure 2 shows the ROC curve based on the time-domain GCM and cGCM measures. Note that there are two plots fewer than the results in the second row since fGCM-Geweke and fGCM-Ent have the same values and fcGCM-Std-Geweke and fcGCM-Std-Ent have the same values. Though cGCM-SEnt still has relatively higher accuracy than the other two cGCM methods, their performance was more similar to the results in the second row of Figure 2. Moreover, the frequency-domain cGCM-SEnt-based results in Figures 2G and 2H have shown better performance than the time-domain measure-based results in Figures 2J and 2K. Results based on the simulation data indicate that the proposed frequency-domain cGCM-SEnt may improve the estimation accuracy of directed connections between time series. For comparison, the results for high-pass filters are provided in the Supporting Information, which also shows a correct selection of the frequency range for ME-based methods that improve the detection of network links.

### Results on rsfMRI Data

**Figure 3.**
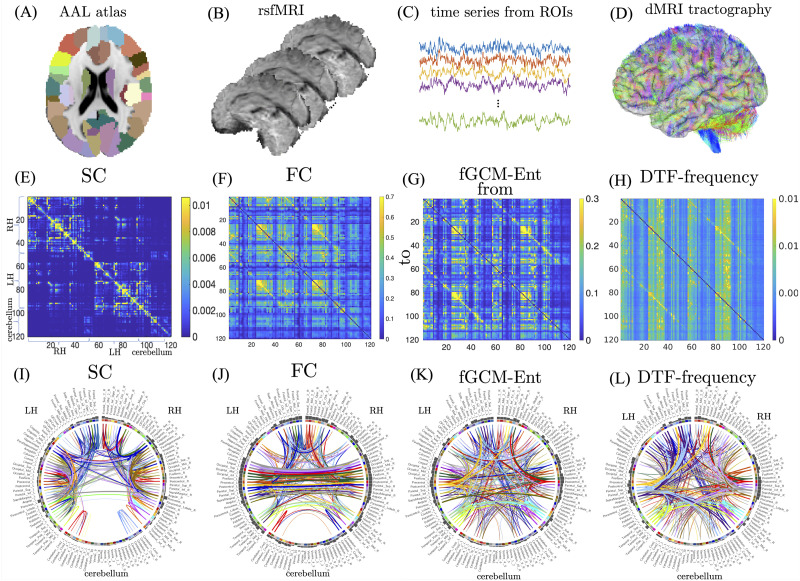
Results of in vivo MRI data analysis. (A) AAL atlas. (B) The rsfMRI volumes. (C) The rsfMRI time series. (D) Whole-brain tractography estimated using dMRI. The second row shows the mean SC, FC, fGCM-Ent, and frequency-domain DTF matrices among 100 subjects. The third row illustrates the brain network connections for the connectivity matrices in the second row.

The first row of Figure 3 briefly summarizes the key experimental procedures, which include the extraction of rsfMRI time series from each region of interest (ROI) based on the AAL atlas and the estimation of structural connectivity matrix based on diffusion MRI tractography.

Figure 3E illustrates the average SC matrix from 100 subjects between 120 ROIs, which are separated into three groups: left-hemisphere (LH), right-hemisphere (RH), and cerebellum as shown in the horizontal and vertical axes. Figure 3F shows the average FC matrix between the 120 ROIs. Figures 3G and 3H show the mean fGCM-Ent and the frequency-domain DTF between 0.01 to 0.1 Hz range related to the hemodynamic response (Biswal, Zerrin Yetkin, Haughton, & Hyde, 1995; Van Dijk et al., 2010). The (*i*, *j*)th entry of the connectivity matrix reflects the causality measures from the *j*th (column) to the *i*th (row) ROI. Figures 3I to 3L in the last row demonstrate the top 400 strongest connections for the connectivity matrices shown in the second row of Figure 3. The sum of the weight of all connections from each brain region gives rise to the node weight, which is shown in black bins.

**Figure 4.**
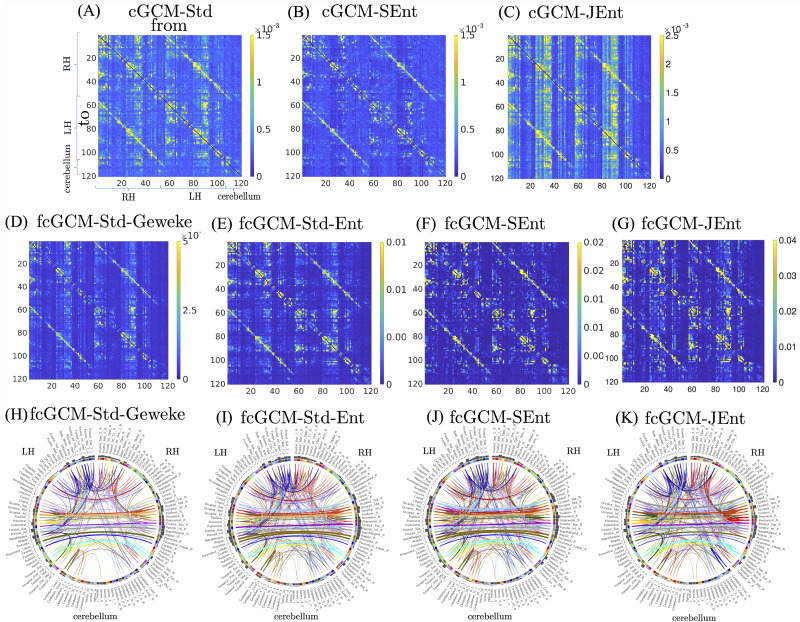
Results of in vivo MRI data analysis. (A) to (C) illustrate the three time-domain cGCM measures. (D) to (G) show the mean fcGCM-Std-Geweke, fcGCM-Std-Ent, fcGCM-SEnt, fcGCM-JEnt between 0.01 and 0.1 Hz among 100 subjects. (H) to (K) illustrate the brain network connections for the connectivity matrices in the second row.

Figure 4 shows the comparisons between different conditional causality measures. In the first row, Figures 4A to 4C show the comparison between the three time-domain cGCM measures. The cGCM-JEnt measure in Figure 4C shows the highest intensity, and the cGCM-SEnt in Figure 4B shows the lowest intensity, which is consistent with Proposition 1. The second row shows the mean values of four fcGCM measures between 0.01 and 0.1 Hz with Figures. 4D to 4G show the results based on standard frequency-domain cGCM (Geweke, 1984), the ME formulation of standard cGCM (Ning & Rathi, 2018), and the proposed separate and joint entropy minimization methods. All four methods show higher values than the corresponding time-domain measures in the first row, indicating stronger causality information contained in the low-frequency range. The third row shows the top 400 strongest connections for the four connectivity matrices in the second row. Different from results in the last row of Figure 3, all four fcGCM methods show connections between symmetric brain regions in the left and right hemispheres, which are not shown in the GCM and DTF methods.

### Correlation Analysis With Structural Connectivity

**Figure 5.**
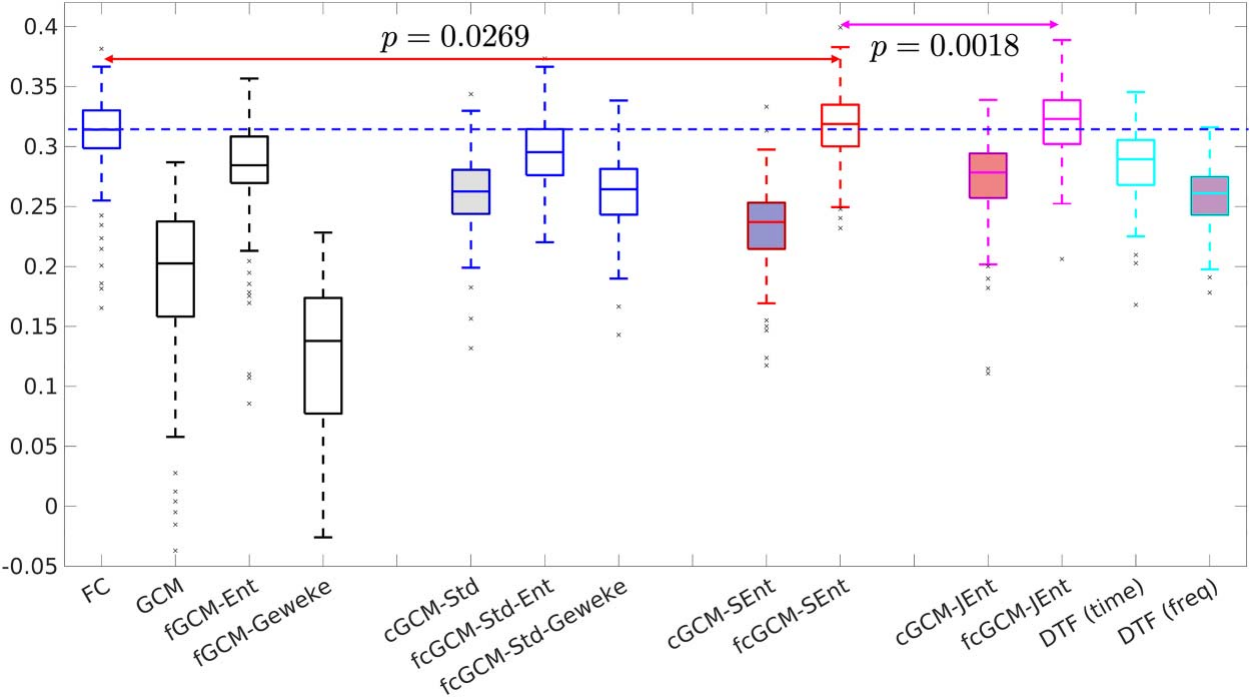
The correlation coefficients between SC and FC, the symmetric part of the GCM, cGCM, and DTF measures in whole-brain networks among 100 subjects. The blue dashed line indicates the mean value of SC-FC correlation coefficients.

Since the ground truth of brain network connections is unknown, the causality measures will be correlated with the SC weights as an approximate form of validation. The plots in Figure 5 illustrate the correlation coefficients between the symmetric SC matrices and the *symmetric* part of the FC-, GCM-, cGCM-, and DTF-based matrices for 100 subjects.

The first plot shows the distribution of the correlation between SC and FC matrices. The blue dashed line indicates the mean value, which is similar to results reported in Fotiadis et al. (2023). The next three plots show the correlation coefficients between SC and the symmetric part of the GCM, the ME-based fGCM-Ent, and the original fGCM-Geweke method, respectively. The fGCM-Ent method has significantly higher (*p* < 10^−60^, *t* test) correlations with SC than the other two methods but with lower values than the results for FC. The following three plots show the standard cGCM method. The fcGCM-Std-Ent method has significantly higher correlation coefficients than the time-domain method cGCM-Std and fcGCM-Std-Geweke but with lower values than the SC-FC correlation coefficients.

The proposed fcGCM-SEnt and fcGCM-JEnt methods all have higher values than the SC-FC correlation coefficients, with the corresponding *p* values from *t* tests shown in the figure. Both methods have higher values than the corresponding time-domain measures and the DTF-based methods as shown in the last two plots. It is noted again that only the symmetric parts of the GCM and cGCM matrices are used in the correlation analysis. Since the SC matrices are symmetric, adding the asymmetric part will reduce the correlation coefficients, as shown in the Supporting Information for completeness.

### Comparison of fGCM and fcGCM

**Figure 6.**
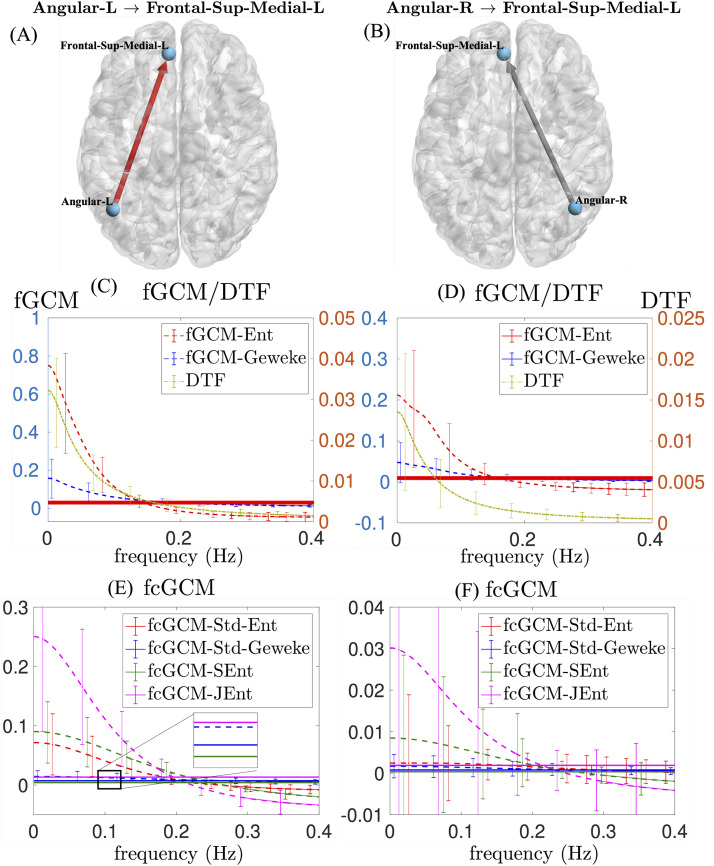
Comparison of the fGCM and fcGCM methods on two brain connections. (A) and (B) illustrate two brain connections. (C) and (D) compare the results of two fGCM methods. (E) and (F) illustrate the results of different fcGCM methods. The mean values of the fGCM and fcGCM measures are shown with solid lines.

To further illustrate the fGCM and fcGCM methods and provide complementary information to Figure 5, Figure 6 shows these frequency-domain measures for two pairs of brain regions. On the left panel, Figures 6C and 6E illustrate the fGCM and fcGCM functions for the connection from left angular (Angular-L) to the left frontal superior medial cortex (Frontal-Sup-Medial-L), which are both involved in the default mode network (Oliver, Hlinka, Kopal, & Davidsen, 2019). The dashed lines illustrate the mean values among 100 subjects, and the error bars show the range of standard deviations. The constant solid lines illustrate the average values of these functions equal to the corresponding time-domain measures. It is noted that the two functions have the same mean.

In Figure 6C, fGCM-Ent (left y-axis) is significantly higher than fGCM-Geweke in the 0.01 to 0.1 Hz frequency range. The DTF functions (right y-axis) have a similar contrast as the fGCM measures. Figure 6E shows the four fcGCM functions of the Angular-L to Frontal-Sup-Medial-L connection, where the three ME-based methods, including fcGCM-Std-Ent, fcGCM-SEnt, and fcGCM-JEnt, all have significantly higher values than the original fcGCM-Std-Geweke method between 0.01 and 0.1 Hz. On the other hand, fcGCM-Std-Geweke shows weaker contrast between different frequencies than other ME-based fcGCM methods. The constant solid lines show the corresponding time-domain measures. The black box shows more details about the difference between the mean values, where fcGCM-JEnt and fcGCM-SEnt have the highest and the lowest values, respectively, consistent with Proposition 1.

The right panel in Figure 6 shows the fGCM and fcGCM functions for the connection from the right angular (Angular-R) to the left frontal superior medial cortex (Frontal-Sup-Medial-L). Since there does not exist anatomically plausible direct structural connections between the two regions, the GCM and cGCM measures are expected to have lower values than the results on the left panel. As expected, the peak magnitudes of fGCM-Ent and fGCM-Geweke in Figure 6D are only 28% and 30% of the corresponding peak magnitudes shown in Figure 6C. In Figure 6F, the peak magnitudes of fcGCM-Std-Ent, fcGCM-Std-Geweke, fcGCM-SEnt, and fcGCM-JEnt are reduced to 3%, 11%, 9%, and 12% of the corresponding peak magnitudes in Figure 6E. More significant reductions in cGCM measures indicate that cGCM performs better in reducing false connections than GCM.

## DISCUSSION

The main contribution of this work is introducing a control-theoretic framework for conditional causal inference for network models of multivariate time series. The proposed method uses causal filters to regress dependence between time series to minimize the entropy rate of the residual process, which is different from the existing transfer entropy (TE)-based interpretation of GCM/cGCM. Three algorithms are developed based on different variations of regression procedures that are equivalent formulations for minimum mean square error (MMSE) estimation methods. The three algorithms provide different frequency-domain formulations to investigate the spectral content network structures. The performance of the time-domain and frequency-domain causality measures are compared using simulations and real neuroimaging data.

The simulation results show that the proposed separate and joint ME methods have improved sensitivity and specificity to detect network connections compared with other GCM/cGCM measures and the direct transfer function (DTF) method. Moreover, the average frequency-domain causality measures in the pass band also improve the detection accuracy than the time-domain measures. Both cGCM-SEnt and cGCM-JEnt have better performance than the standard cGCM and FC methods in the correlation analysis with the SC matrices. It is interesting to note that SC-FC correlation coefficients are higher than the results for the standard GCM and cGCM methods. The cGCM-JEnt method had a slightly stronger correlation with SC than the cGCM-SEnt method. The improved performance for cGCM-JEnt may be related to the fact that the JEnt method uses relatively conservative but optimal ME filters to regress out ***z*** from the joint process (***x***; ***y***), which is helpful to enhance the performance for causal inference.

Results based on real functional MRI data have shown consistent results that the proposed joint and separate entropy minimization methods outperform other methods to detect effective connections that are correlated with the structural brain networks. The frequency-domain measures provide significantly higher correlation coefficients with the structural connectivity than the time-domain measures. Thus, the proposed method can potentially be a useful tool in neuroimaging analysis. The toolbox used to compute the proposed measures is available at GitHub (https://github.com/LipengNing/ME-GCM). Below are some discussions that highlight the limitations and future work.

The proposed method is limited by the assumptions of linear models and stationary Gaussian processes. Linear systems cannot accurately model biological measurements (Friston et al., 2013; Jirsa, Jantzen, Fuchs, & Kelso, 2002; Valdes-Sosa, Roebroeck, Daunizeau, & Friston, 2011; Valdes-Sosa et al., 2009) or time-varying dynamics in brain networks (Liu & Duyn, 2013; Ning, 2019, 2020; Zalesky, Fornito, Cocchi, Gollo, & Breakspear, 2014). Generalizations of the ME method for nonlinear, non-Gaussian, or nonstationary processes and the nonparametric method for ME estimation will be derived in future work. Moreover, the proposed solutions for the ME filters are derived based on the assumption that the diagonal entries of the VAR-type representations are stably invertible. Although this assumption is generally satisfied in real fMRI data, more general solutions will also need to be developed.

Further validations are needed for the real-data example. There are other factors, such as heterogeneous and non-minimum-phase hemodynamic response functions (Solo, 2016), that may impact the causality measures. Application of the proposed method to other image modalities, such as EEG and MEG, may provide more direct information about neural activities and richer spectral content to cross-validate results from this study. Another limitation of the real-data example is that the ground truth is unknown, and the SC measures are used for an approximated form of validation. However, the SC matrix may be biased or have missing connections for brain network modeling. Deco et al. (2014) showed that adding more cross-hemispheric connections and reweighing existing connections improve the correlation between structural and functional connectivity. Moreover, the selected AAL atlas may not provide all relevant brain regions in functional terms for brain network analysis. Thus, further validation can be done by examining the correlation between causality measures and SC with additional links. Furthermore, this work used random permutation testing as in Barnett and Seth (2014) to examine the performance of causality measures. The computational complexity is a limitation for applications in large-scale networks. Moreover, the random permutation does not preserve the autocorrelation of the time series. Thus, more effective statistical testing methods are needed in further work.

Finally, it is noted that there are several other methods derived for causal inference, including the modified GCM method (Hosoya, Oya, Takimoto, & Kinoshita, 2017) and the generalized partial directed coherence (Baccala, Sameshima, & Takahashi, 2007). A comprehensive comparison of these methods is beyond the scope of this paper and will be explored in future work.

## CONCLUSIONS

The proposed ME-based methods for conditional causality analysis, especially cGCM-SEnt, provide more sensitive and specific measures than the standard methods in detecting network connections. The ME-based frequency-domain methods can correctly characterize the band-pass property of brain activities using neuroimaging data. Thus, the ME-based method can provide more effective tools for causal inference of direct connections in networks, which may be useful for neuroimaging analysis. Future work will focus on deriving the general solution for ME filters without the assumption of stable diagonal blocks of VAR models and the development of efficient computation algorithms and statistical analysis methods. Moreover, the integration of structural constraints in model estimation will be explored in future work to reduce redundant parameters and improve the reliability of the proposed measures for large-scale time series analysis. Furthermore, nonlinear generalization of the proposed measures as in Keskin and Aste (2020), Marinazzo et al. (2011), Seth and Príncipe (2012), and Sun (2008) will also be explored in future work.

## ACKNOWLEDGMENTS

This work has been supported by the following grants funded by the National Institutes of Health: R21MH115280 (PI: Ning), R21MH116352 (PI: Ning), K01MH117346 (PI: Ning), R01MH132610 (PI: O’Donnell, Makris, Rathi), R01MH125860 (PI: O’Donnell, Makris, Rathi), R61MH132869 (PI: Camprodon).

## SUPPORTING INFORMATION

Supporting information for this article is available at https://doi.org/10.1162/netn_a_00386.

## AUTHOR CONTRIBUTIONS

Lipeng Ning: Conceptualization; Data curation; Formal analysis; Funding acquisition; Investigation; Methodology; Project administration; Resources; Software; Validation; Visualization; Writing – original draft; Writing – review & editing.

## FUNDING INFORMATION

Lipeng Ning, National Institute of Mental Health (https://dx.doi.org/10.13039/100000025), Award ID: R21MH116352, R21MH126396, R21MH115280, K01MH117346.

## Supplementary Material



## TECHNICAL TERMS

Granger causality measure (GCM): A scalar value that assesses whether past values of one time series can predict future values in another, indicating potential causation.
Conditional Granger causality measure (cGCM): An extension of GCM by considering causation between time series while accounting for the influence of additional variables.
Shannon entropy: A measure that quantifies information content in a random variable or dataset, measuring the average surprise or unpredictability.
State-space representation: A mathematical modeling method that describes dynamic systems using state variables and equations to predict their behavior and future states.
Spectral factorization: A mathematical process to break down a signal’s spectrum into constituent parts for analysis or processing.
