# Supplementary material for: An information-theoretic framework for conditional causality analysis of brain networks
Source: Netw Neurosci. 2024 Oct 1;8(3):989–1008. doi: 10.1162/netn_a_00386 (PMC11424036; doi:10.1162/netn_a_00386)
Supplement: Supplementary file 1 [file netn-8-3-989-s001.pdf]

Journal: **NETWORK NEUROSCIENCE**

METHODS

An information-theoretic framework for conditional causality analysis of brain networks: Supplemental Materials

Lipeng Ning[1,2]

[1]Brigham and Women's Hospital, Boston, MA, USA

[2]Harvard Medical School, Boston, MA, USA

STATE-SPACE REPRESENTATION AND SPECTRAL FACTORIZATION

A wide-sense stationary process $\{\boldsymbol{u}_t\}$ for $\boldsymbol{u}_t \in \mathbb{R}^n$ can be approximated by a vector autoregressive and moving average (VARMA) model using the following state space representation

$$\boldsymbol{\xi}_{t+1} = A\boldsymbol{\xi}_t + B\boldsymbol{\epsilon}_t, \tag{S.1a}$$

$$\boldsymbol{u}_t = C\boldsymbol{\xi}_t + \boldsymbol{\epsilon}_t, \tag{S.1b}$$

where $A \in \mathbb{R}^{m\times m}, B \in \mathbb{R}^{m\times n}, C \in \mathbb{R}^{n\times m}$ with $m \geq n$ and $\boldsymbol{\epsilon} \in \mathbb{R}^n$ represents the zero-mean white Gaussian innovation process with $\mathcal{E}(\boldsymbol{\epsilon}\boldsymbol{\epsilon}^T) = \Omega$. Moreover, the VARMA representation can be transformed into an innovative form using the spectral factorization algorithm so that the transfer function $H(L)$ from $\boldsymbol{\epsilon}_t$ to $\boldsymbol{u}_t$ is minimum-phase and $H(0) = I$ (Kailath, Sayed, & Hassibi, 2000; Wiener & Masani, 1957).

Consider the following general SSR for the joint process $\boldsymbol{u}_t = [\boldsymbol{x}_t; \boldsymbol{y}_t; \boldsymbol{z}_t]$

$$\boldsymbol{\xi}_{t+1} = A\boldsymbol{\xi}_t + \boldsymbol{\zeta}_t, \tag{S.2a}$$

$$\boldsymbol{u}_t = C\boldsymbol{\xi}_t + \boldsymbol{\eta}_t, \tag{S.2b}$$

where $\boldsymbol{\zeta}_t \in \mathbb{R}^m$ and $\boldsymbol{\eta}_t \in \mathbb{R}^n$ with $m \geq n$ are zero-mean white Gaussian processes with the following covariance matrix

$$\mathcal{E}\left(\begin{bmatrix} \boldsymbol{\zeta}_t\boldsymbol{\zeta}_t^T & \boldsymbol{\zeta}_t\boldsymbol{v}_t^T \\ \boldsymbol{\eta}_t\boldsymbol{\zeta}_t^T & \boldsymbol{\eta}_t\boldsymbol{v}_t^T \end{bmatrix}\right) = \begin{bmatrix} Q & S \\ S^T & R \end{bmatrix}. \tag{S.3}$$

The spectral factorization algorithm can be used to transform (S.2) to an innovation form as in (S.1) (Kailath et al., 2000). The corresponding matrices B and Ω in (S.1) are computed by

$$\Omega = CPC^T + R \tag{S.4}$$

$$B = (APC^T + S)\Omega^{-1}, \tag{S.5}$$

where P is the solution of the following discrete-time algebraic Riccati equation (DARE):

$$P - APA^T = Q - (APC^T + S)(CPC^T + R)^{-1}(CPA^T + S^T). \tag{S.6}$$

The proposed methods depend on the spectral factorization for (S.13) and (S.64), which can be represented in the form of (S.2) with suitable choices of model parameters that are derived from the joint models of the multivariate time series.

Based on the spectral factorization algorithm, the power spectral density (PSD) function of $\boldsymbol{u}_t$ can be expressed as

$$S(\theta) = H(e^{i\theta})\Omega H(e^{i\theta})^*, \tag{S.7}$$

where $i = \sqrt{-1}$, and

$$H(L) = I_n + C(I_m - AL)^{-1}BL \tag{S.8}$$

represents the transfer function from $\boldsymbol{\epsilon}$ to $\boldsymbol{u}$ such that

$$\boldsymbol{u} = H(L)\boldsymbol{\epsilon}. \tag{S.9}$$

Moreover, based on the spectral factorization, $H(L)$ is a minimum-phase transfer function, implying all its zeros are outside of the unit circle. For convenience, $H(L)$ is represented by

$$H(L) \sim \left(\begin{array}{c|c} A & B \\ \hline C & I_n \end{array}\right). \tag{S.10}$$

The inverse representation of (S.9) is given by the following "vector autoregressive (VAR)-type" representation

$$G(L)\boldsymbol{u} = \boldsymbol{\epsilon},, \tag{S.11}$$

with $G(L) = H(L)^{-1}$ whose state-space representation is given by

$$G(L) \sim \left(\begin{array}{c|c} A - BC & B \\ \hline -C & I_n \end{array}\right). \tag{S.12}$$

We note that the term "VAR-type" indicates that $G(L)$ is formally the inverse of $H(L)$, which can be considered as a vector moving average (VMA) representation. Thus, $G(L)$ may be resented by a VARMA or an infinite VAR model. If $H(L)$ is a minimum-phase spectral factorization, then $A - BC$ is stable, i.e., the eigenvalues of $A - BC$ are within the unit circle. Throughout the paper, all the diagonal blocks of $G(L)$ and other VAR-type filters are assumed to have stable inversion. Specifically, it is assumed that $A - \sum_{k\in\mathcal{K}} b_k c_k^T$ is stable for all subsets $\mathcal{K} \subseteq \{1, \ldots, n\}$ with b_k and c_k being the k-th columns of B and C^T, respectively.

Based on (S.9), the joint process $\{\boldsymbol{x}; \boldsymbol{y}\}$ is represented by

$$\begin{bmatrix} \boldsymbol{x} \\ \boldsymbol{y} \end{bmatrix} = \begin{bmatrix} I_{n_x+ny} & 0 \end{bmatrix} H(L)\boldsymbol{\epsilon}, \tag{S.13}$$

which can be converted to the following representation using the spectral factorization algorithm

$$\begin{bmatrix} \boldsymbol{x} \\ \boldsymbol{y} \end{bmatrix} = \hat{H}(L) \begin{bmatrix} \hat{\boldsymbol{\epsilon}}_x \\ \hat{\boldsymbol{\epsilon}}_y \end{bmatrix}. \tag{S.14}$$

The spectral factorization algorithm requires the solution to a discrete-time algebraic Riccati equation (DARE), which can be obtained by using an iteration algorithm with an $o(n)$ complexity in each iteration

(Chu & Weng, 2015) for large-scale systems. Let $\hat{G}(L) = \hat{H}(L)^{-1}$ whose state-space representation can be derived using (S.12). Then the VAR representation for $\{\boldsymbol{x}; \boldsymbol{z}\}$ is given by (4). The ME filter $F(L)$ that minimizes the entropy of $\boldsymbol{x}(t) - F(L)\boldsymbol{y}(t)$ is given by

$$F(L) = -\hat{G}_{xx}^{-1}(L)\hat{G}_{xy}(L). \tag{S.15}$$

The ME residual process is given by

$$\boldsymbol{x}||\boldsymbol{y}_t = \boldsymbol{x}_t - F(L)\boldsymbol{y}_t \tag{S.16}$$

$$= \hat{G}_{xx}^{-1}(L)\tilde{\boldsymbol{\epsilon}}_{x,t}. \tag{S.17}$$

The PSD function $\boldsymbol{x}||\boldsymbol{y}$ is given by

$$S_{\boldsymbol{x}||\boldsymbol{y}}(\theta) = \hat{G}_{xx}^{-1}(e^{i\theta})\hat{\Omega}_{xx}\hat{G}_{xx}^{-*}(e^{i\theta}). \tag{S.18}$$

The entropy rate of the process $\{\boldsymbol{x}||\boldsymbol{y}_t\}$ is equal to (Kailath et al., 2000; Ning & Rathi, 2018; Wiener & Masani, 1957).

$$h(\{\boldsymbol{x}||\boldsymbol{y}\}) = \frac{1}{4\pi}\int_{-\pi}^{\pi} \ln\det S_{\boldsymbol{x}||\boldsymbol{y}}(\theta)d\theta + \frac{1}{2}n_x(1+\ln(2\pi)), \tag{S.19}$$

$$= \frac{1}{2}\ln\det\hat{\Omega}_{xx} + \frac{1}{2}n_x(1+\ln(2\pi)). \tag{S.20}$$

The subprocess $\{\boldsymbol{x}_t\}$ can be extracted from the joint process and represented by

$$\boldsymbol{x}_t = [I_{n_x}\ 0]H(L)\boldsymbol{\epsilon}_t. \tag{S.21}$$

Based on the similar procedure as in (S.13) to (S.18), the spectral factorization algorithm can be applied to compute the PSD function of $\boldsymbol{x}$ as

$$S_x(\theta) = A_{xx}(e^{i\theta})^{-1}\Sigma_{xx}A_{xx}(e^{i\theta})^{-*}, \tag{S.22}$$

as in (7) of the manuscript. Then, the entropy rate of the process $\{\boldsymbol{x}_t\}$ is equal to

$$h(\{\boldsymbol{x}\}) = \frac{1}{2}\ln\det\Sigma_{xx} + \frac{1}{2}n_x(1+\ln(2\pi)), \tag{S.23}$$

according to (8) in the main manuscript.

ME-BASED FORMULATION OF GCM

It was shown in (Ning & Rathi, 2018) that the GCM from $\boldsymbol{y}$ to $\boldsymbol{x}$ is related to the entropy difference between $\boldsymbol{x}$ and $\boldsymbol{x}||\boldsymbol{y}$ as

$$\mathcal{F}_{\boldsymbol{y}\to\boldsymbol{x}} = 2(h(\{\boldsymbol{x}\}) - h(\{\boldsymbol{x}||\boldsymbol{y}\})), \tag{S.24}$$

$$= \ln \frac{\det \Sigma_{xx}}{\det \hat{\Omega}_{xx}}. \tag{S.25}$$

The ME-based frequency-domain formulation for GCM (Ning & Rathi, 2018) is defined as

$$\mathrm{f}^{\mathrm{Ent}}_{\boldsymbol{y}\to\boldsymbol{x}}(\theta) = \ln \frac{\det S_{\boldsymbol{x}}(\theta)}{\det S_{\boldsymbol{x}||\boldsymbol{y}}(\theta)}. \tag{S.26}$$

Then, the following equation holds

$$\mathcal{F}_{\boldsymbol{y}\to\boldsymbol{x}} = \frac{1}{2\pi}\int_{-\pi}^{\pi} \mathrm{f}^{\mathrm{Ent}}_{\boldsymbol{y}\to\boldsymbol{x}}(\theta) d\theta, \tag{S.27}$$

which shows that the time-domain GCM $\mathcal{F}^{\mathrm{Std}}_{\boldsymbol{y}\to\boldsymbol{x}}$ equals the average value of the frequency-domain measures.

The GCM between other pairs of time series can be derived by following the same methods as in the computation of $\mathcal{F}^{\mathrm{Std}}_{\boldsymbol{y}\to\boldsymbol{x}}$. Thus, for an n-dimensional time series, the power spectral factorization is needed for $\frac{n(n-1)}{2}$ pairs of time series and each of the n subprocesses.

ME-BASED FORMULATION OF CGCM AND THE STANDARD METHOD

To introduce the ME-based cGCM $\mathcal{F}_{\boldsymbol{y}\to\boldsymbol{x}|\boldsymbol{z}}$ in (Ning & Rathi, 2018), we first consider the following VAR model of the joint process $\{\boldsymbol{x};\boldsymbol{z}\}$

$$\begin{bmatrix} \tilde{G}_{xx}(L) & \tilde{G}_{xz}(L) \\ \tilde{G}_{zx}(L) & \tilde{G}_{zz}(L) \end{bmatrix} \begin{bmatrix} \boldsymbol{x} \\ \boldsymbol{z} \end{bmatrix} = \begin{bmatrix} \tilde{\boldsymbol{\epsilon}}_x \\ \tilde{\boldsymbol{\epsilon}}_z \end{bmatrix}, \tag{S.28}$$

with

$$\mathcal{E}\left(\begin{bmatrix} \tilde{\boldsymbol{\epsilon}}_x \\ \tilde{\boldsymbol{\epsilon}}_z \end{bmatrix} \begin{bmatrix} \tilde{\boldsymbol{\epsilon}}_x^T & \tilde{\boldsymbol{\epsilon}}_z^T \end{bmatrix} \right) = \begin{bmatrix} \tilde{\Omega}_{xx} & \tilde{\Omega}_{xz} \\ \tilde{\Omega}_{zx} & \tilde{\Omega}_{zz} \end{bmatrix}. \tag{S.29}$$

Based on (S.15) to (S.18), the ME residual $\boldsymbol{x}||\boldsymbol{z}$ is equal to

$$\boldsymbol{x}||\boldsymbol{z}_t = \tilde{G}_{xx}^{-1}(L)\tilde{\boldsymbol{\epsilon}}_{x,t}, \tag{S.30}$$

whose entropy and PSD functions are equal to

$$h(\{\boldsymbol{x}||\boldsymbol{z}\}) = \frac{1}{2}\ln\det\tilde{\Omega}_{xx} + \frac{1}{2}n_x(1+\ln(2\pi)), \tag{S.31}$$

$$S_{\boldsymbol{x}||\boldsymbol{z}}(\theta) = \tilde{G}_{xx}^{-1}(e^{i\theta})\tilde{\Omega}_{xx}\tilde{G}_{xx}^{-*}(e^{i\theta}), \tag{S.32}$$

respectively.

Based on the VAR model for the joint process $\{\boldsymbol{x};\boldsymbol{y};\boldsymbol{z}\}$ as shown in (1), the ME process $\boldsymbol{x}||\boldsymbol{y}\boldsymbol{z}$ is given by

$$\boldsymbol{x}||\boldsymbol{y}\boldsymbol{z} = G_{xx}^{-1}(L)\boldsymbol{\epsilon}_x, \tag{S.33}$$

whose entropy and PSD function are equal to

$$h(\{\boldsymbol{x}||\boldsymbol{y}\boldsymbol{z}\}) = \frac{1}{2}\ln\det\Omega_{xx} + \frac{1}{2}n_x(1+\ln(2\pi)) \tag{S.34}$$

$$S_{\boldsymbol{x}||\boldsymbol{y}\boldsymbol{z}}(\theta) = G_{xx}^{-1}(e^{i\theta})\Omega_{xx}G_{xx}^{-*}(e^{i\theta}). \tag{S.35}$$

The standard cGCM method, denoted by cGCM-Std, can be expressed using the ME process as

$$\mathcal{F}^{\text{Std}}_{\boldsymbol{y}\to\boldsymbol{x}|\boldsymbol{z}} = 2(h(\{\boldsymbol{x}||\boldsymbol{z}\}) - h(\{\boldsymbol{x}||\boldsymbol{y}\boldsymbol{z}\})), \tag{S.36}$$

$$= \log\frac{\det\tilde{\Omega}_{xx}}{\det\Omega_{xx}}. \tag{S.37}$$

The corresponding frequency-domain formulation is defined as

$$\mathrm{f}^{\text{Std}-\text{Ent}}_{\boldsymbol{y}\to\boldsymbol{x}|\boldsymbol{z}}(\theta) = \ln\frac{\det S_{\boldsymbol{x}||\boldsymbol{z}}(\theta)}{\det S_{\boldsymbol{x}||\boldsymbol{y}\boldsymbol{z}}(\theta)}, \tag{S.38}$$

whose mean value is equal to $\mathcal{F}^{\text{Std}}_{\boldsymbol{y}\to\boldsymbol{x}|\boldsymbol{z}}$.

For comparison, the original fcGCM, referred to as fcGCM-Std-Geweke, is introduced below. First, combine (S.28) and (S.9) to obtain the following equations

$$\begin{bmatrix}\tilde{\boldsymbol{\epsilon}}_x\\ \boldsymbol{y}\\ \tilde{\boldsymbol{\epsilon}}_z\end{bmatrix} = \begin{bmatrix}\tilde{G}_{xx}(L) & 0 & \tilde{G}_{xz}(L)\\ 0 & I_{n_y} & 0\\ \tilde{G}_{zx}(L) & 0 & \tilde{G}_{zz}(L)\end{bmatrix} \times \begin{bmatrix}H_{xx}(L) & H_{xy}(L) & H_{xz}(L)\\ H_{yx}(L) & H_{yy}(L) & H_{yz}(L)\\ H_{zx}(L) & H_{zy}(L) & H_{zz}(L)\end{bmatrix}\begin{bmatrix}\boldsymbol{\epsilon}_x\\ \boldsymbol{\epsilon}_y\\ \boldsymbol{\epsilon}_z\end{bmatrix}, \tag{S.39}$$

$$= \begin{bmatrix}P_{xx}(L) & P_{xy}(L) & P_{xz}(L)\\ P_{yx}(L) & P_{yy}(L) & P_{yz}(L)\\ P_{zx}(L) & P_{zy}(L) & P_{zz}(L)\end{bmatrix}\begin{bmatrix}\boldsymbol{\epsilon}_x\\ \boldsymbol{\epsilon}_y\\ \boldsymbol{\epsilon}_z\end{bmatrix}. \tag{S.40}$$

The original fcGCM can be computed as

$$\mathrm{f}^{\mathrm{Std-Geweke}}_{\boldsymbol{y}\to\boldsymbol{x}|\boldsymbol{z}}(\theta) = \mathrm{f}^{\mathrm{Geweke}}_{\tilde{\boldsymbol{\epsilon}}_z\boldsymbol{y}\to\tilde{\boldsymbol{\epsilon}}_x}(\theta), \tag{S.41}$$

$$= \ln\frac{\det\tilde{\Omega}_{xx}}{\det(\tilde{\Omega}_{xx} - [P_{xy}(e^{i\theta})\; P_{xz}(e^{i\theta})]\Omega_{yz|x}[P_{xy}(e^{i\theta})\; P_{xz}(e^{i\theta})]^*)}, \tag{S.42}$$

where

$$\Omega_{yz|x} = \begin{bmatrix}\Omega_{yy} & \Omega_{yz}\\ \Omega_{zy} & \Omega_{zz}\end{bmatrix} - \begin{bmatrix}\Omega_{yx}\\ \Omega_{zx}\end{bmatrix}\Omega_{xx}^{-1}\begin{bmatrix}\Omega_{xy} & \Omega_{xz}\end{bmatrix}. \tag{S.43}$$

The mean value of $\mathrm{f}^{\mathrm{Std-Geweke}}_{\boldsymbol{y}\to\boldsymbol{x}|\boldsymbol{z}}(\theta)$ is equal to $\mathcal{F}^{\mathrm{Std}}_{\boldsymbol{y}\to\boldsymbol{x}|\boldsymbol{z}}$ based on (S.20).

SEPARATE ME-BASED CGCM

This subsection introduces the computational algorithms for cGCM-SEnt and cGCM-JEnt in Figs. 1(d) and 1(e). Assume the joint process $(\boldsymbol{y};\boldsymbol{z})$ is represented by

$$\begin{bmatrix}\check{G}_{yy}(L) & \check{G}_{yz}(L)\\ \check{G}_{zy}(L) & \check{G}_{zz}(L)\end{bmatrix}\begin{bmatrix}\boldsymbol{y}\\ \boldsymbol{z}\end{bmatrix} = \begin{bmatrix}\check{\boldsymbol{\epsilon}}_y\\ \check{\boldsymbol{\epsilon}}_z\end{bmatrix}, \tag{S.44}$$

where

$$\mathcal{E}\left(\begin{bmatrix}\check{\boldsymbol{\epsilon}}_y\\ \check{\boldsymbol{\epsilon}}_z\end{bmatrix}\begin{bmatrix}\check{\boldsymbol{\epsilon}}_y^T & \check{\boldsymbol{\epsilon}}_z^T\end{bmatrix}\right)=\begin{bmatrix}\check{\Omega}_{yy} & \check{\Omega}_{yz}\\ \check{\Omega}_{zy} & \check{\Omega}_{zz}\end{bmatrix}. \tag{S.45}$$

The ME process $\boldsymbol{y}||\boldsymbol{z}$ is given by

$$\boldsymbol{y}||\boldsymbol{z}=\check{G}_{yy}(L)^{-1}\check{\boldsymbol{\epsilon}}_y. \tag{S.46}$$

By definition, cGCM-SEnt and fcGCM-SEnt are equal to

$$\mathcal{F}^{\text{SEnt}}_{\boldsymbol{y}\to\boldsymbol{x}||\boldsymbol{z}}=\mathcal{F}_{\boldsymbol{y}||\boldsymbol{z}\to\boldsymbol{x}||\boldsymbol{z}}, \tag{S.47}$$

$$\text{f}^{\text{SEnt}}_{\boldsymbol{y}\to\boldsymbol{x}||\boldsymbol{z}}(\theta)=\text{f}_{\boldsymbol{y}||\boldsymbol{z}\to\boldsymbol{x}||\boldsymbol{z}}(\theta), \tag{S.48}$$

respectively. The following lemma is useful to derive the computation methods for $\mathcal{F}^{\text{SEnt}}_{\boldsymbol{y}\to\boldsymbol{x}||\boldsymbol{z}}$ and $\text{f}^{\text{SEnt}}_{\boldsymbol{y}\to\boldsymbol{x}||\boldsymbol{z}}(\theta)$ which will be useful to prove Proposition 2.

Lemma 1. *For $\tilde{\boldsymbol{\epsilon}}_x$ and $\check{\boldsymbol{\epsilon}}_y$ defined in* (S.28) *and* (S.44)*, respectively, the following equations hold.*

$$\mathcal{F}_{\boldsymbol{y}||\boldsymbol{z}\to\boldsymbol{x}||\boldsymbol{z}}=\mathcal{F}_{\check{\boldsymbol{\epsilon}}_y\to\tilde{\boldsymbol{\epsilon}}_x}, \tag{S.49}$$

$$\text{f}_{\boldsymbol{y}||\boldsymbol{z}\to\boldsymbol{x}||\boldsymbol{z}}(\theta)=\text{f}_{\check{\boldsymbol{\epsilon}}_y\to\tilde{\boldsymbol{\epsilon}}_x}(\theta). \tag{S.50}$$

Proof. Let $\hat{F}(L)$ denote the causal filter that minimizes the entropy of

$$\hat{\boldsymbol{x}}||\boldsymbol{y}\boldsymbol{z}=\boldsymbol{x}||\boldsymbol{z}-\hat{F}(L)\boldsymbol{y}||\boldsymbol{z}. \tag{S.51}$$

From (S.30) and (S.46), the above equation is equal to

$$\hat{\boldsymbol{x}}||\boldsymbol{y}\boldsymbol{z}=\tilde{G}_{xx}(L)^{-1}\left(\tilde{\boldsymbol{\epsilon}}_x-\tilde{G}_{xx}(L)\hat{F}(L)\check{G}_{yy}(L)^{-1}\check{\boldsymbol{\epsilon}}_y\right). \tag{S.52}$$

Therefore, the entropy of the process $\hat{\boldsymbol{x}}||\boldsymbol{y}\boldsymbol{z}$ is equal to that of $\tilde{\boldsymbol{\epsilon}}_x-\tilde{G}_{xx}(L)\hat{F}(L)\check{G}_{yy}(L)^{-1}\check{\boldsymbol{\epsilon}}_y$.

On the other hand, if $\tilde{F}(L)$ is a causal filter that minimizes the entropy of

$$\tilde{\boldsymbol{\epsilon}}_x||\check{\boldsymbol{\epsilon}}_y:=\tilde{\boldsymbol{\epsilon}}_x-\tilde{F}(L)\boldsymbol{\epsilon}_y, \tag{S.53}$$

then setting

$$\hat{F}(L) = \tilde{G}_{xx}(L)^{-1}\tilde{F}(L)\check{G}_{yy}(L) \tag{S.54}$$

minimizes the entropy of $\hat{\boldsymbol{x}}||\boldsymbol{yz}$. Thus,

$$\hat{\boldsymbol{x}}||\boldsymbol{yz} = \tilde{G}_{xx}(L)^{-1}\tilde{\boldsymbol{\epsilon}}_x||\check{\boldsymbol{\epsilon}}_y, \tag{S.55}$$

which implies

$$h(\{\hat{\boldsymbol{x}}||\boldsymbol{yz}\}) = h(\{\tilde{\boldsymbol{\epsilon}}_x||\check{\boldsymbol{\epsilon}}_y\}). \tag{S.56}$$

On the other hand, (S.30) imply that

$$h(\{\boldsymbol{x}||\boldsymbol{z}\}) = h(\{\tilde{\boldsymbol{\epsilon}}_x\}). \tag{S.57}$$

Thus,

$$\mathcal{F}_{\boldsymbol{y}||\boldsymbol{z}\to\boldsymbol{x}||\boldsymbol{z}} = h(\{\boldsymbol{x}||\boldsymbol{z}\}) - h(\{\hat{\boldsymbol{x}}||\boldsymbol{yz}\}) \tag{S.58}$$

$$= h(\{\tilde{\boldsymbol{\epsilon}}_x\}) - h(\{\tilde{\boldsymbol{\epsilon}}_x||\check{\boldsymbol{\epsilon}}_y\}), \tag{S.59}$$

which proves (S.49).

To prove (S.50), we note that

$$\det S_{\boldsymbol{x}||\boldsymbol{z}}(\theta) = \det(\tilde{G}_{xx}^{-1}(e^{i\theta})\tilde{G}_{xx}^{-*}(e^{i\theta}))\det\tilde{\Omega}_{xx}, \tag{S.60}$$

$$\det S_{\hat{\boldsymbol{x}}||\boldsymbol{yz}}(\theta) = \det(\tilde{G}_{xx}^{-1}(e^{i\theta})\tilde{G}_{xx}^{-*}(e^{i\theta}))\det S_{\tilde{\boldsymbol{\epsilon}}_x||\check{\boldsymbol{\epsilon}}_y}(\theta). \tag{S.61}$$

Therefore, the following equations hold.

$$\begin{aligned}
\mathrm{f}_{\boldsymbol{y}||\boldsymbol{z}\to\boldsymbol{x}||\boldsymbol{z}}(\theta) &= \ln\frac{\det S_{\boldsymbol{y}||\boldsymbol{z}}(\theta)}{\det S_{\hat{\boldsymbol{x}}||\boldsymbol{yz}}(\theta)}\\
&= \ln\frac{\det\tilde{\Omega}_{xx}}{\det S_{\tilde{\boldsymbol{\epsilon}}_x||\check{\boldsymbol{\epsilon}}_y}(\theta)}\\
&= \mathrm{f}_{\check{\boldsymbol{\epsilon}}_y\to\tilde{\boldsymbol{\epsilon}}_x}(\theta),
\end{aligned}$$

which completes the proof. □

Based on Lemma 1, the computation for $\mathcal{F}^{\mathrm{SEnt}}_{\boldsymbol{y}\to\boldsymbol{x}|\boldsymbol{z}}$ requires a joint model for $\check{\boldsymbol{\epsilon}}_y; \tilde{\boldsymbol{\epsilon}}_x$. To this end, the SSR of the joint process $\tilde{\boldsymbol{\epsilon}}_x; \check{\boldsymbol{\epsilon}}_y$ is derived below. The following matrices are introduced to derive the joint model

$$B_a = [\tilde{B}_x, 0, \tilde{B}_z], \tag{S.62}$$

$$B_b = [0, \check{B}_y, \check{B}_z], \tag{S.63}$$

where $\tilde{B}$ and $\check{B}$ are the corresponding input matrices of the SSR of the innovation form for $\tilde{G}(L)$ in (S.28) and $\check{G}(L)$ in (S.44), respectively. $\tilde{B}_x$ and $\tilde{B}_z$ represent the first n_x and the last n_z columns of $\tilde{B}$, $\check{B}_y$ and $\check{B}_z$ represent the first n_y and last n_z columns of $\check{B}$, respectively. By multiplying the transfer function from $\boldsymbol{u}_t$ to $\tilde{\boldsymbol{\epsilon}}_x$ and $\check{\boldsymbol{\epsilon}}_y$ and the transfer function from $\boldsymbol{\epsilon}_t$ to $\boldsymbol{u}_t$, the transfer function from $\boldsymbol{\epsilon}_t$ to $\tilde{\boldsymbol{\epsilon}}_x$ and $\check{\boldsymbol{\epsilon}}_y$ can be computed as

$$H^{\mathrm{aug}}(L) \sim \left(\begin{array}{c|c} A^{\mathrm{aug}} & B^{\mathrm{aug}} \\ \hline C^{\mathrm{aug}} & D^{\mathrm{aug}} \end{array}\right), \tag{S.64}$$

where

$$A^{\mathrm{aug}} = \begin{bmatrix} A & 0 & 0 \\ B_a C & A - \tilde{B}\tilde{C} & 0 \\ B_b C & 0 & A - \check{B}\check{C} \end{bmatrix}, \tag{S.65}$$

$$B^{\mathrm{aug}} = [B;\ B_a;\ B_b]\,, \tag{S.66}$$

$$C^{\mathrm{aug}} = \begin{bmatrix} C_x & -C_x & 0 \\ C_y & -C_y & 0 \end{bmatrix}, \tag{S.67}$$

$$D^{\mathrm{aug}} = [I_{n_x+n_y}\ 0], \tag{S.68}$$

where C_x and C_y are the first n_x rows and the following n_y rows of the measurement matrix C. Next, $\mathcal{F}_{\boldsymbol{y}||\boldsymbol{z}\to\boldsymbol{x}||\boldsymbol{z}}$ and $\mathrm{f}_{\check{\epsilon}_y\to\tilde{\epsilon}_x}(\theta)$ can be computed by using the spectral factorization algorithm.

JOINT ME-BASED CGCM

To compute the cGCM-JEnt measure illustrated in Fig.1(e), consider the following representation $(\boldsymbol{x}^{\text{JEnt}}||\boldsymbol{z}; \boldsymbol{y}^{\text{JEnt}}||\boldsymbol{z})$

$$\begin{bmatrix} \boldsymbol{x}^{\text{JEnt}}||\boldsymbol{z} \\ \boldsymbol{y}^{\text{JEnt}}||\boldsymbol{z} \end{bmatrix} = \begin{bmatrix} G_{xx}(L) & G_{xy}(L) \\ G_{yx}(L) & G_{yy}(L) \end{bmatrix}^{-1} \begin{bmatrix} \boldsymbol{\epsilon}_x \\ \boldsymbol{\epsilon}_y \end{bmatrix}. \tag{S.69}$$

The SSR of the above transfer function is given by

$$\begin{bmatrix} G_{xx}(L) & G_{xy}(L) \\ G_{yx}(L) & G_{yy}(L) \end{bmatrix}^{-1} \sim \left(\begin{array}{c|c} A - B_z C_z & [B_x \ B_y] \\ \hline \begin{bmatrix} C_x \\ C_y \end{bmatrix} & I_{n_x+n_y} \end{array} \right), \tag{S.70}$$

where $B = [B_x, B_y, B_z]$ and $C^T = [C_x^T, C_y^T, C_z^T]$.

Moreover, the ME process obtained by regressing the past values of $\boldsymbol{y}^{\text{JEnt}}||\boldsymbol{z}$ from $\boldsymbol{x}^{\text{JEnt}}||\boldsymbol{z}$ is equal to

$$\boldsymbol{x}^{\text{JEnt}}||\boldsymbol{yz} = \boldsymbol{x}^{\text{JEnt}}||\boldsymbol{z} + G_{xx}(L)^{-1} G_{xy}(L) \boldsymbol{y}^{\text{JEnt}}||\boldsymbol{z}, \tag{S.71}$$

$$= G_{xx}(L)^{-1} \boldsymbol{\epsilon}_x. \tag{S.72}$$

Thus the PSD function of $\boldsymbol{x}^{\text{JEnt}}||\boldsymbol{yz}$ is given by

$$S_{\boldsymbol{x}^{\text{JEnt}}||\boldsymbol{yz}}(\theta) = G_{xx}^{-1}(e^{i\theta}) \Omega_{xx} G_{xx}^{-*}(e^{i\theta}). \tag{S.73}$$

On the other hand, the PSD function of $\boldsymbol{x}^{\text{JEnt}}||\boldsymbol{z}$ is equal to

$$S_{\boldsymbol{x}^{\text{JEnt}}||\boldsymbol{z}}(\theta) = [I_{n_x}, \ 0] \begin{bmatrix} G_{xx}(e^{i\theta}) & G_{xy}(e^{i\theta}) \\ G_{yx}(e^{i\theta}) & G_{yy}(e^{i\theta}) \end{bmatrix}^{-1} \begin{bmatrix} \Omega_{xx} & \Omega_{xy} \\ \Omega_{yx} & \Omega_{yy} \end{bmatrix} \times \begin{bmatrix} G_{xx}(e^{i\theta}) & G_{xy}(e^{i\theta}) \\ G_{yx}(e^{i\theta}) & G_{yy}(e^{i\theta}) \end{bmatrix}^{-*} \begin{bmatrix} I_{n_x} \\ 0 \end{bmatrix}. \tag{S.74}$$

By using the SSR in (S.70) and the spectral factorization algorithm, $S_{\boldsymbol{x}^{\text{JEnt}}||\boldsymbol{yz}}(\theta)$ can be factorized as

$$S_{\boldsymbol{x}^{\text{JEnt}}||\boldsymbol{z}}(\theta) = P_{x^{\text{JEnt}}||z}(e^{i\theta}) \Sigma_{x^{\text{JEnt}}||z} P_{x^{\text{JEnt}}||z}(e^{i\theta})^*, \tag{S.75}$$

where $P_{x^{\rm JEnt}||z}(e^{i\theta})$ is the unique minimum-phase spectral factor. Then, cGCM-JEnt and fcGCM-JEnt are given by

$$\begin{aligned}\mathcal{F}^{\rm JEnt}_{\boldsymbol{y}\to\boldsymbol{x}||\boldsymbol{z}} &= 2(h(\{\boldsymbol{x}^{\rm JEnt}||\boldsymbol{z}\}) - h(\{\boldsymbol{x}^{\rm JEnt}||\boldsymbol{yz}\})),\\ &= \ln\frac{\det\Sigma_{x^{\rm JEnt}||z}}{\det\Omega_{xx}}, \qquad\qquad \text{(S.76)}\\ {\rm f}^{\rm JEnt}_{\boldsymbol{y}\to\boldsymbol{x}||\boldsymbol{z}}(\theta) &= \ln\frac{\det S_{\boldsymbol{x}^{\rm JEnt}||\boldsymbol{z}}(\theta)}{\det S_{\boldsymbol{x}^{\rm JEnt}||\boldsymbol{yz}}(\theta)}, \qquad\qquad \text{(S.77)}\end{aligned}$$

respectively, and the mean value of ${\rm f}^{\rm JEnt}_{\boldsymbol{y}\to\boldsymbol{x}||\boldsymbol{z}}(\theta)$ is equal to $\mathcal{F}^{\rm JEnt}_{\boldsymbol{y}\to\boldsymbol{x}||\boldsymbol{z}}$.

It is noted that ${\rm f}^{\rm SEnt}_{\boldsymbol{y}\to\boldsymbol{x}||\boldsymbol{z}}$ has the highest computational complexity among the three proposed cGCM methods since it involves a spectral factorization of the augmented system in (S.64). Both the ME-based fcGCM ${\rm f}^{\rm Std-Ent}_{\boldsymbol{y}\to\boldsymbol{x}|\boldsymbol{z}}$ and the original ${\rm f}^{\rm Std-Geweke}_{\boldsymbol{y}\to\boldsymbol{x}|\boldsymbol{z}}$ methods have similar computational complexity since they all involve calculation of the representation for the joint process $(\boldsymbol{x};\boldsymbol{z})$ by using the spectral factorization algorithm. The joint ME method ${\rm f}^{\rm JEnt}_{\boldsymbol{y}\to\boldsymbol{x}||\boldsymbol{z}}$ has the least computational complexity since all the variables are directly provided by the VAR-type model. Though an accurate solution for the corresponding time-domain measure $\mathcal{F}^{\rm JEnt}_{\boldsymbol{y}\to\boldsymbol{x}||\boldsymbol{z}}$ still requires a spectral factorization algorithm to obtain $\det\Sigma_{x^{\rm JEnt}||z}$, an approximate value for $\det\Sigma_{x^{\rm JEnt}||z}$ can be obtained by a discrete approximation for the geometric mean of $\det S_{\boldsymbol{x}^{\rm JEnt}||\boldsymbol{z}}(\theta)$.

PROOF OF PROPOSITION 1

Proof. Based on the definitions of $\boldsymbol{x}^{\rm JEnt}||\boldsymbol{yz}$ and $\boldsymbol{x}||\boldsymbol{yz}$, it can be shown that

$$\boldsymbol{x}^{\rm JEnt}||\boldsymbol{yz} = \boldsymbol{x}||\boldsymbol{yz} = G_{xx}^{-1}(L)\boldsymbol{\epsilon}_x, \qquad \text{(S.78)}$$

where more details can be found in (S.33) and (S.72) in Appendix. Since $\boldsymbol{x}||\boldsymbol{z}$ is the ME estimator for $\boldsymbol{x}$ using the past values of $\boldsymbol{z}$ and $\boldsymbol{x}^{\rm JEnt}||\boldsymbol{z}$ involves entropy minimization of the joint process $(\boldsymbol{x};\boldsymbol{y})$ based on a joint model of $(\boldsymbol{x};\boldsymbol{y};\boldsymbol{z})$, it holds that

$$h(\{\boldsymbol{x}^{\rm JEnt}||\boldsymbol{z}\}) \geq h(\{\boldsymbol{x}||\boldsymbol{z}\}). \qquad \text{(S.79)}$$

Combining (S.78) and (S.79) leads to

$$\begin{aligned}\mathcal{F}^{\text{JEnt}}_{\boldsymbol{y}\to\boldsymbol{x}|\boldsymbol{z}} &= 2(h(\{\boldsymbol{x}^{\text{JEnt}}||\boldsymbol{z}\}) - h(\{\boldsymbol{x}^{\text{JEnt}}||\boldsymbol{yz}\})),\\ &\geq 2(h(\{\boldsymbol{x}||\boldsymbol{z}\}) - h(\{\boldsymbol{x}^{\text{JEnt}}||\boldsymbol{yz}\})),\\ &= \mathcal{F}^{\text{Std}}_{\boldsymbol{y}\to\boldsymbol{x}|\boldsymbol{z}}.\end{aligned} \tag{S.80}$$

Furthermore, since $\boldsymbol{x}||\boldsymbol{yz}$ is the ME estimator for $\boldsymbol{x}$ using the joint process $\boldsymbol{y}$ and $\boldsymbol{z}$, it holds that

$$h(\{\boldsymbol{x}^{\text{SEnt}}||\boldsymbol{yz}\}) \geq h(\{\boldsymbol{x}||\boldsymbol{yz}\}). \tag{S.81}$$

Therefore, the following inequality holds

$$\begin{aligned}\mathcal{F}^{\text{Std}}_{\boldsymbol{y}\to\boldsymbol{x}|\boldsymbol{z}} &= 2(h(\{\boldsymbol{x}||\boldsymbol{z}\}) - h(\{\boldsymbol{x}||\boldsymbol{yz}\})),\\ &\geq 2(h(\{\boldsymbol{x}||\boldsymbol{z}\}) - h(\{\boldsymbol{x}^{\text{SEnt}}||\boldsymbol{yz}\})),\\ &= \mathcal{F}^{\text{SEnt}}_{\boldsymbol{y}\to\boldsymbol{x}|\boldsymbol{z}}.\end{aligned} \tag{S.82}$$

Moreover, by definition, $\mathcal{F}^{\text{SEnt}}_{\boldsymbol{y}\to\boldsymbol{x}|\boldsymbol{z}} \geq 0$ which completes the proof. □

PROOF OF PROPOSITION 2

Proof. Based on the definition, it follows that if $G_{xy}(L) = 0$ then

$$\boldsymbol{x}^{\text{JEnt}}||\boldsymbol{z} = G^{-1}_{xx}(L)\boldsymbol{\epsilon}_x,$$

which is equal to $\boldsymbol{x}^{\text{JEnt}}||\boldsymbol{yz}$. Therefore, $\mathcal{F}^{\text{JEnt}}_{\boldsymbol{y}\to\boldsymbol{x}|\boldsymbol{z}} = 0$. Thus, Proposition 1 indicates that $\mathcal{F}^{\text{JEnt}}_{\boldsymbol{y}\to\boldsymbol{x}|\boldsymbol{z}} = \mathcal{F}^{\text{Std}}_{\boldsymbol{y}\to\boldsymbol{x}|\boldsymbol{z}} = \mathcal{F}^{\text{SEnt}}_{\boldsymbol{y}\to\boldsymbol{x}|\boldsymbol{z}} = 0$. It remains to show that if $G_{xy}(L) \neq 0$, then

$$\mathcal{F}^{\text{SEnt}}_{\boldsymbol{y}\to\boldsymbol{x}|\boldsymbol{z}} > 0. \tag{S.83}$$

For this purpose, consider the following representation for the joint processes $(\boldsymbol{x};\boldsymbol{z})$ and $(\boldsymbol{y};\boldsymbol{z})$

$$\tilde{G}_{xx}(L)\boldsymbol{x} + \tilde{G}_{xz}(L)\boldsymbol{z} = \tilde{\boldsymbol{\epsilon}}_x, \tag{S.84}$$

$$\check{G}_{yy}(L)\boldsymbol{y} + \check{G}_{yz}(L)\boldsymbol{z} = \check{\boldsymbol{\epsilon}}_y. \tag{S.85}$$

Note that $\boldsymbol{x}||\boldsymbol{z} = \tilde{G}_{xx}(L)^{-1}\tilde{\boldsymbol{\epsilon}}_x$ and $\boldsymbol{y}||\boldsymbol{z} = \check{G}_{yy}(L)^{-1}\check{\boldsymbol{\epsilon}}_y$. Lemma 1 shows that $\mathcal{F}^{\text{SEnt}}_{\boldsymbol{y}\to\boldsymbol{x}|\boldsymbol{z}} = \mathcal{F}_{\check{\boldsymbol{\epsilon}}_y\to\tilde{\boldsymbol{\epsilon}}_x}$. Therefore, it is equivalent to prove that if $G_{xy}(L) \neq 0$ then $\mathcal{F}_{\check{\boldsymbol{\epsilon}}_y\to\tilde{\boldsymbol{\epsilon}}_x} > 0$.

Assume that $\mathcal{F}_{\check{\boldsymbol{\epsilon}}_y \to \tilde{\boldsymbol{\epsilon}}_x} = 0$. Then it indicates that $\tilde{\boldsymbol{\epsilon}}_x$ is the innovation process for the joint modeling of $(\tilde{\boldsymbol{\epsilon}}_x; \check{\boldsymbol{\epsilon}}_y)$ since the past values of $\check{\boldsymbol{\epsilon}}_y$ do not improve the prediction of $\tilde{\boldsymbol{\epsilon}}_x$. Thus, $\tilde{\boldsymbol{\epsilon}}_{x,t}$ is uncorrelated with the past values of $\check{\epsilon}_{y,t}$ which is denoted by $\tilde{\boldsymbol{\epsilon}}_x \perp H\{\check{\epsilon}_{y,t-k}, k \in \mathbb{Z}^+\}$ where $H\{\check{\boldsymbol{\epsilon}}_{y,t-k}, k \in \mathbb{Z}^+\}$ denotes the Hilbert space spanned by all the past variables of $\check{\boldsymbol{\epsilon}}_y$ based on the standard inner product. By definition $\tilde{\boldsymbol{\epsilon}}_x$ satisfies that $\tilde{\boldsymbol{\epsilon}}_x \perp H\{\boldsymbol{z}_{t-k}, k \in \mathbb{Z}^+\}$ and $\tilde{\boldsymbol{\epsilon}}_x \perp H\{\boldsymbol{x}_{t-k}, k \in \mathbb{Z}^+\}$ since $\tilde{\boldsymbol{\epsilon}}_x$ is the innovation process in (S.84). Then,

$$\mathcal{E}(F(L)\hat{\boldsymbol{\epsilon}}_y \tilde{\boldsymbol{\epsilon}}_x^T) = \mathcal{E}(F(L)(\hat{G}_{yx}(L)\boldsymbol{x} + \hat{G}_{yy}(L)\boldsymbol{y})\tilde{\boldsymbol{\epsilon}}_x^T) \tag{S.86}$$

$$= \mathcal{E}(F(L)\hat{G}_{yy}(L)\boldsymbol{y}\tilde{\boldsymbol{\epsilon}}_x^T) = 0, \tag{S.87}$$

for any causal filter $F(L)$ because $\mathcal{E}(\hat{G}_{yx}(L)\boldsymbol{x}\tilde{\boldsymbol{\epsilon}}_x^T) = 0$. Since $\hat{G}_{yy}(L)$ is invertible by assumption, then

$$\tilde{\boldsymbol{\epsilon}}_x \perp H\{\boldsymbol{y}_{t-k}, k \in \mathbb{Z}^+\}.$$

Therefore, $\tilde{\boldsymbol{\epsilon}}_x \perp H\{\boldsymbol{x}_{t-k}, \boldsymbol{y}_{t-k}, \boldsymbol{z}_{t-k}, k \in \mathbb{Z}^+\}$ which indicates that $\tilde{\boldsymbol{\epsilon}}_x = \boldsymbol{\epsilon}_x$. Thus, (S.84) implies that the optimal $G_{xy}(L) = 0$, which contradicts the assumption that $G_{xy}(L) \neq 0$. Therefore, $\mathcal{F}_{\check{\boldsymbol{\epsilon}}_y \to \tilde{\boldsymbol{\epsilon}}_x} > 0$ which completes the proof. □

ADDITIONAL SIMULATION RESULTS

Experiment based on two-node models

Fig. S1 illustrates an example with two-node networks to show the different performance of ME-based, standard GCM, and DTF methods. Similar to the simulation experiment in the paper, each link in Fig. S1 represents a coefficient of the VAR model. The coefficient is set to positive or negative values to simulate low-pass and high-pass filters.

The second row of Fig. S1 shows the results for low-pass filters. Fig. S1(b) compares the frequency-domain GCM-Orig, GCM-Eng, and DTF functions. Note that the cGCM measures are not needed for a two-node system. Figs. S1(c) - (d) show ROC curves based on the mean measures for frequencies in $[0, \frac{\pi}{2}]$, and $[0, \pi]$, respectively. The interval $[0, \frac{\pi}{2}]$ is selected heuristically to examine if low-frequency measures improve the detection of network connections since the VAR model is low-pass. Note that the mean values for GCM-Ent and GCM-Orig in $[0, \pi]$ are the same. Therefore, GCM and DTF are shown in Fig. S1(d). The GCM-Ent method has a much higher true positive rate using measures

in $[0,\ \frac{\pi}{2}]$ than results based on $[0,\ \pi]$, indicating the frequency-domain GCM-Ent in the pass band of the VAR filters is useful to improve the detection of network links. On the other hand, the DTF and GCM-Orig methods have similar performance over different frequency ranges.

The third row of Fig. S1 shows the results for high-pass filters. Fig. S1(e) shows the frequency-domain GCM-Orig, GCM-Eng, and DTF functions. Figs. S1(f) - (g) show ROC curves based on the mean measures for frequencies in $[\frac{\pi}{2},\ \pi]$, and $[0,\ \pi]$, respectively. Note that the GCM-Ent method has a much higher true positive rate based on frequencies in $[\frac{\pi}{2},\ \pi]$ than the results based on $[0,\ \pi]$ since the filter is high-pass. On the other hand, the DTF and GCM-Orig methods have similar performance over different frequency ranges, indicating reduced specificity in the frequency domain for network detections.

Experiment based on high-pass filters

Fig. S2 provides complementary information to the simulation experiments, as shown in Fig. 2 of the paper, on the performance of the methods for high-pass filters. The first row of Fig. Fig. S2 shows the same network structures as in the paper but with negative model parameters to simulate high-pass VAR filters. The second row of Fig. S2 shows the frequency-domain GCM, cGCM, and DTF measures, which all have higher values in the high-frequency range close π and the low-frequency values.

The last three rows show the ROC plots based on the mean values in the $[\frac{\pi}{2},\ \pi]$ and $[0,\ \pi]$ range, respectively. Figs. S2(g) - (i) show that the cGCM-SENt and cGCM-JEnt in the high-frequency range have higher true positive rates than other methods but with much worse performance in the low-frequency-domain. The three cGCM methods have similar results based on the average measures in the $[0,\ \pi]$ and are better than the GCM and DTF methods. Thus, the results in Fig. S2 further confirm the results shown in the paper that the ME-based cGCM methods provide frequency-specific information on brain connectivity.

REFERENCES

Chu, E. K. W., & Weng, P. C. Y. (2015). Large-scale discrete-time algebraic Riccati equations - Doubling algorithm and error analysis. *Journal of Computational and Applied Mathematics*, *277*, 115–126. Retrieved from

http://dx.doi.org/10.1016/j.cam.2014.09.005 doi: 10.1016/j.cam.2014.09.005

Hamilton. (1993). Time series analysis / James D. Hamilton. *Time series analysis*.

Kailath, T., Sayed, A., & Hassibi, B. (2000). *Linear Estimation*. Upper Saddle River, NJ.: Prentice Hall.

Ning, L., & Rathi, Y. (2018). A Dynamic Regression Approach for Frequency-Domain Partial Coherence and Causality Analysis of Functional Brain Networks. *IEEE Transactions on Medical Imaging*. doi: 10.1109/TMI.2017.2739740

Wiener, N., & Masani, P. (1957). The prediction theory of multivariate stochastic processes, Part I. *Acta Math.*, *98*, 111–150.

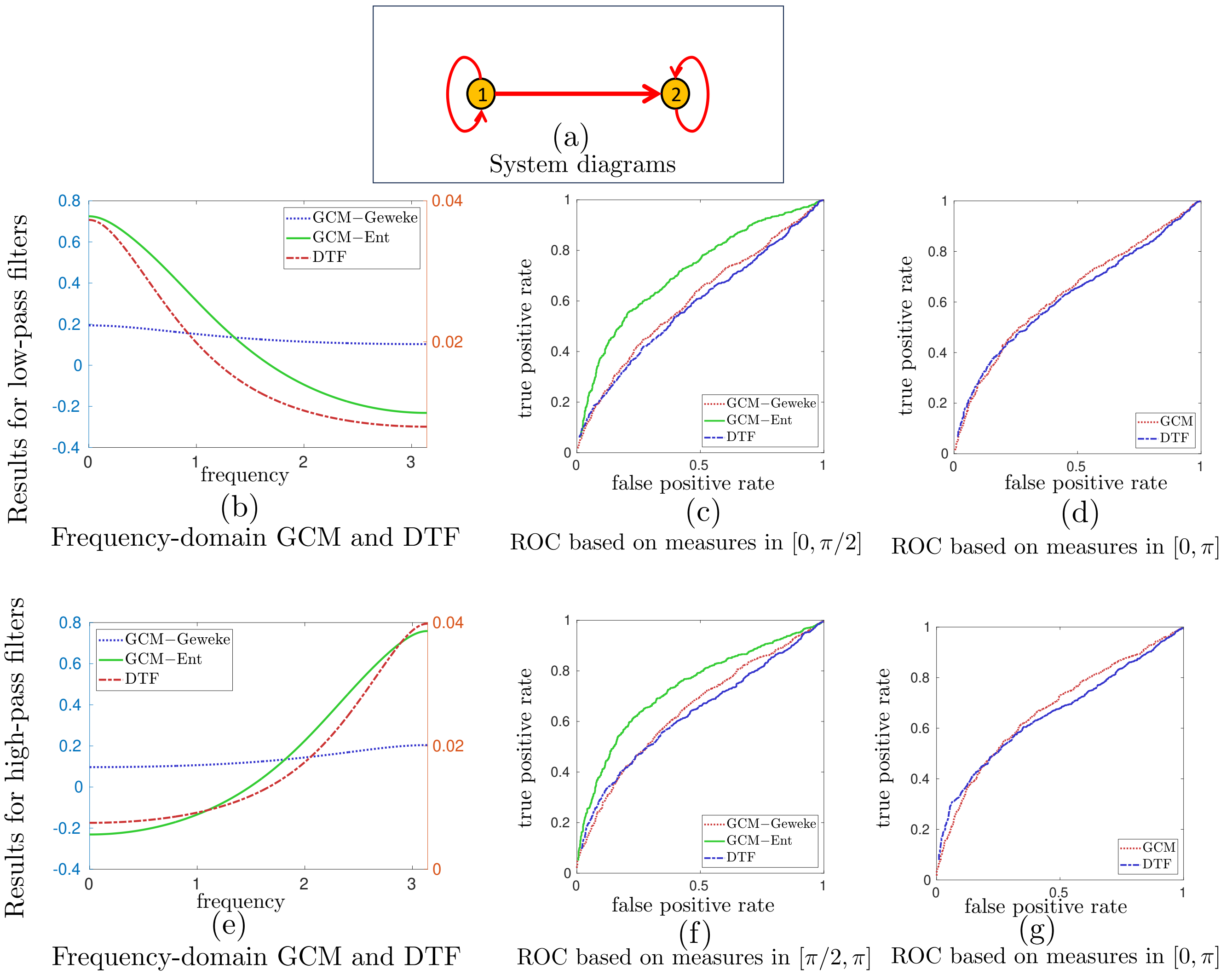

178 **Figure S1.** Simulation results for two two-node networks as shown in (a) with low- and high-pass VAR filters. (b) shows the frequency-domain GCM-Orig,
179 GCM-Ent, and DTF measures for the low-pass filter. (c) to (d) show the ROC plots corresponding to mean measures in $[0, \frac{\pi}{2}]$ and $[0, \pi]$, respectively. (e) -
180 (f) illustrate the measures for the high-pass filter and the ROC curves based on measures in $[\frac{\pi}{2}, \pi]$ and $[0, \pi]$, respectively.

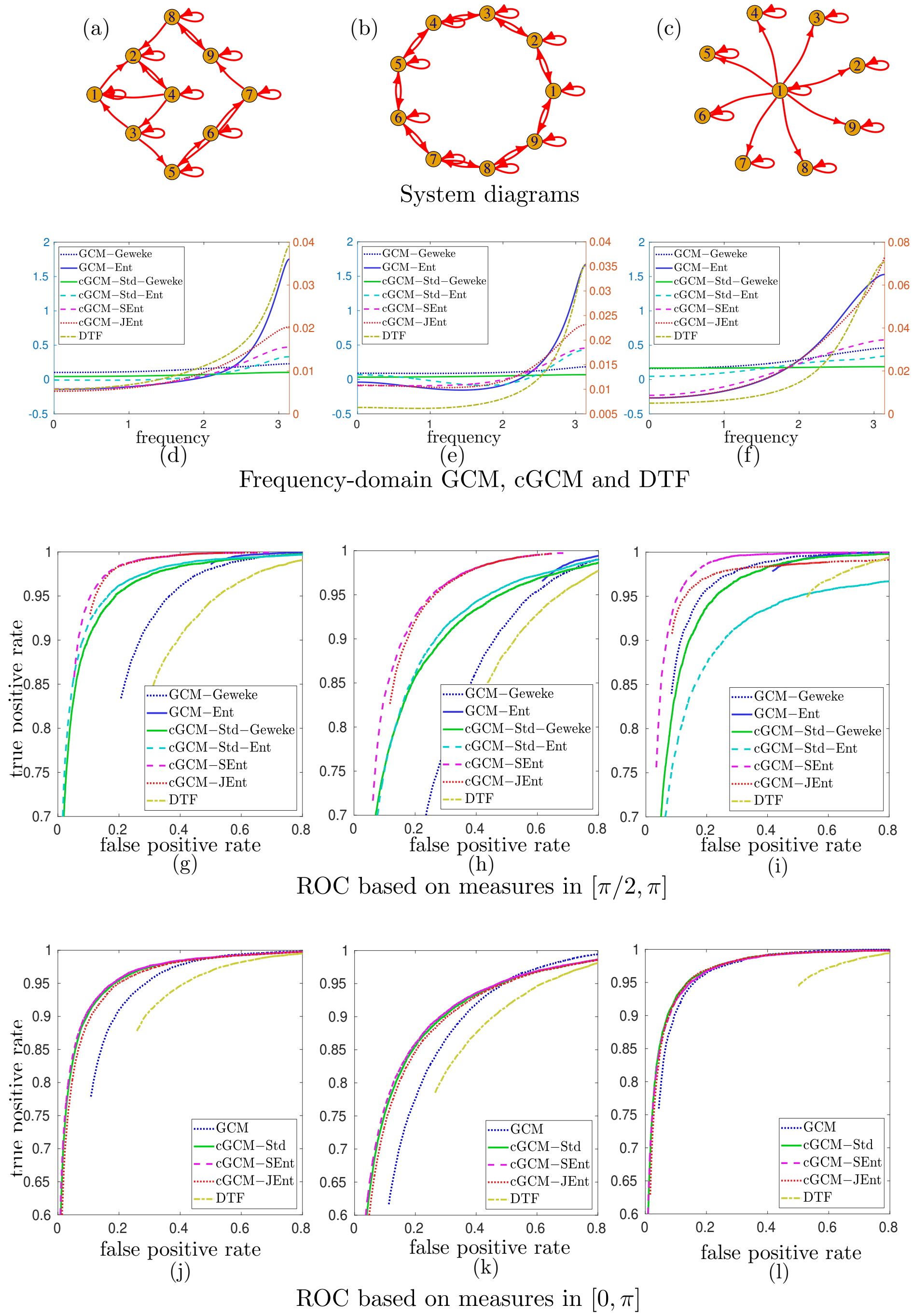

Figure S2. Illustration of simulation results corresponding to high-pass VAR models. The first row demonstrates the structure of three VAR-type models used in the simulations. The second row shows the sample mean of frequency-domain GCM, cGCM, and DTF functions for all non-zero connections in the first row. The last three rows show the ROC curves based on the mean values of the causality measures for frequencies in $[\frac{\pi}{2},\ \pi]$ and $[0,\ \pi]$, respectively.
